# MorphoSeq: Full Single-Cell Transcriptome Dynamics Up to Gastrulation in a Chordate

**DOI:** 10.1016/j.cell.2020.03.055

**Published:** 2020-05-14

**Authors:** Hanna L. Sladitschek, Ulla-Maj Fiuza, Dinko Pavlinic, Vladimir Benes, Lars Hufnagel, Pierre A. Neveu

**Affiliations:** 1 Cell Biology and Biophysics Unit, European Molecular Biology Laboratory, 69117 Heidelberg, Germany; 2 Department of Molecular Medicine, University of Padua School of Medicine, 35126 Padua, Italy; 3 Genomics Core Facility, European Molecular Biology Laboratory, 69117 Heidelberg, Germany

**Keywords:** single-cell RNA sequencing, light sheet imaging, ascidian, gene expression noise, cell type classification, spatial reconstruction, lineage reconstruction, embryogenesis, cell fate specification

## Abstract

Single-cell RNA sequencing (scRNA-seq) provides a leap forward in resolving cellular diversity and developmental trajectories but fails to comprehensively delineate the spatial organization and precise cellular makeup of individual embryos. Here, we reconstruct from scRNA-seq and light sheet imaging data a canonical digital embryo that captures the genome-wide gene expression trajectory of every single cell at every cell division in the 18 lineages up to gastrulation in the ascidian *Phallusia mammillata*. By using high-coverage scRNA-seq, we devise a computational framework that stratifies single cells of individual embryos into cell types without prior knowledge. Unbiased transcriptome data analysis mapped each cell’s physical position and lineage history, yielding the complete history of gene expression at the genome-wide level for every single cell in a developing embryo. A comparison of individual embryos reveals both extensive reproducibility between symmetric embryo sides and a large inter-embryonic variability due to small differences in embryogenesis timing.

## Highlights

• Integration of scRNA-seq and imaging data yield a canonical digital embryo
• Cell type classification without prior knowledge
• *De novo* reconstruction of the lineage history and spatial organization of the embryo
• Timing differences contribute to inter-embryo variability in gene expression

## Graphical Abstract

**Figure:**
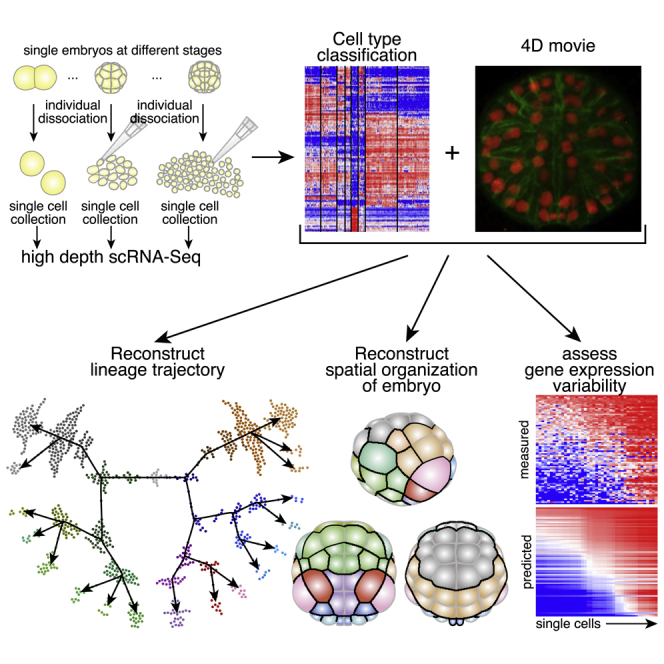

## Teaser

The complete gene expression history and physical position of every single cell at every cell division in an embryo up to gastrulation is reconstructed using a combination of single-cell transcriptomics and light sheet imaging.

## Introduction

One of the most fundamental problems of biology is how the many different cell types of a multicellular organism are generated from a single-celled fertilized egg during embryonic development. Studying the developmental mechanisms driving this cellular diversification at the single-cell, genome-wide, and whole-embryo level has remained challenging. So far, most studies have focused on either a limited number of marker genes or a few select cell lineages. Consequently, we currently lack a comprehensive understanding of the gene expression programs that instruct individual cells to acquire all the cell fates necessary to build an embryo.

Although the advent of single-cell transcriptomics was a major advance to stratify organisms into different cell populations (Cao et al., 2017, Karaiskos et al., 2017), the information of where individual cells originally came from as well as their past or future expression trajectory are typically lost. Indeed, current approaches rely on prior knowledge to reconstruct the spatial organization of embryos (Satija et al., 2015, Achim et al., 2015) or physically sectioning the embryos (Combs and Eisen, 2013, Junker et al., 2014, Peng et al., 2019). Although complex spatial gene expression patterns have been inferred from single-cell RNA sequencing (scRNA-seq) with limited prior information (Karaiskos et al., 2017), a recent seminal study demonstrated that such *de novo* reconstructions do not necessarily rely on prior knowledge (Nitzan et al., 2019). Assessing different developmental stages enabled the reconstruction of lineage histories (Scialdone et al., 2016, Telley et al., 2016, Tusi et al., 2018, Rosenberg et al., 2018, Briggs et al., 2018, Wagner et al., 2018, Farrell et al., 2018, Cao et al., 2019). Despite these recent advances, an *in toto* representation of embryonic development accounting for every single cell in space and time has not been achieved.

**Figure 1:**
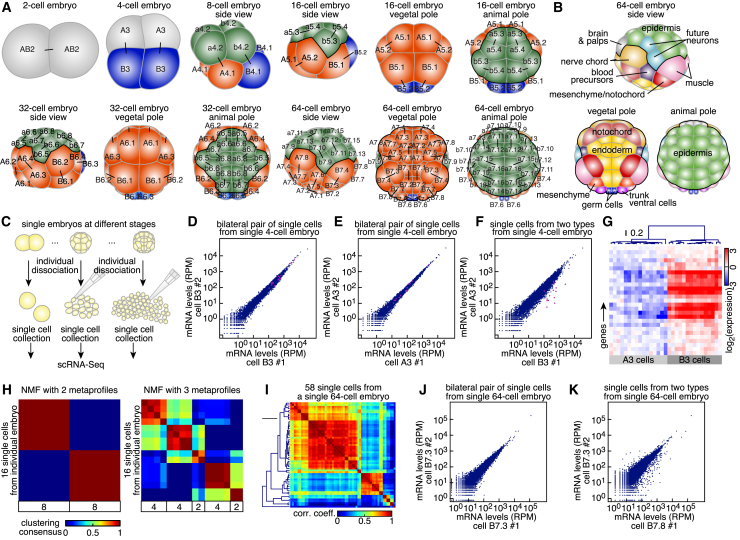
scRNA-Seq Captures the Bilateral Symmetry of the *P. mammillata* Embryo (A) Scheme of *P. mammillata* embryos up to the 64-cell stage. Cell labeling according to Conklin (1905). Blue, germ cell lineage; green, animal (ectoderm) pole; orange, somatic cells of the vegetal (endoderm and mesoderm) pole. Bars link sister cells. (B) Fates of individual cells at the 64-cell stage. (C) Experimental principle to capture expression profiles of cells from a single embryo. (D–F) scRNA-seq analysis of the two cell pairs ([D] and [E]) or of two cells belonging to each cell pair (F) from the same 4-cell embryo (magenta, asymmetrically apportioned maternal factors). (G) Expression levels of the 27 identified maternal factors that were asymmetrically apportioned in single cells of 4-cell embryos. (H) Non-negative matrix factorization of gene expression profiles of all 16 cells of a 16-cell embryo. (I) Hierarchical clustering of 58 single-cell gene expression profiles of a same 64-cell embryo. (J and K) scRNA-seq analysis of one bilateral cell pair (J) or two cells belonging to two different cell pairs (K) from the same 64-cell embryo. See also Figure S1.

To unbiasedly reconstruct embryonic development from scRNA-seq data, we exploited the advantageous properties of the chordate *Phallusia mammillata* (Conklin, 1905, Zalokar and Sardet, 1984). This ascidian combines the genomic complexity and embryonic cell diversity of a vertebrate with a relatively small total number of cells stereotypically segregating into lineages (Figure 1A) in an optically transparent embryo (Corbo et al., 2001). Indeed, cell fates of the future nerve chord, brain, germ cells, blood precursors, and muscles are already specified at the 64-cell stage (Nishida, 1987) (Figure 1B), at which point endoderm cells begin to deform to initiate gastrulation (Sherrard et al., 2010).

Making use of the stereotypic chordate development, we combine high-resolution single-cell transcriptomics and light sheet imaging to generate a comprehensive four-dimensional (4D) atlas of embryonic gene expression in every cell for each cell division up to gastrulation in *P. mammillata*. By using high-coverage scRNA-seq, we devised a computational framework that identifies cell types within individual embryos without prior knowledge. Moreover, we designed methods that unbiasedly infer the spatial coordinates and mother-daughter relatedness of every embryonic cell directly from their transcriptome data. The integration and quantification of *in toto* cell shape reconstructions from 4D imaging and scRNA-seq data uncovered the patterned expression of specific protocadherins. Comparing high-resolution gene expression datasets from individual embryos revealed both extensive reproducibility between the bilaterally symmetric embryo sides and a large degree of inter-embryonic variability. We anticipate that the digital chordate embryo we report here will be a rich resource to mine the molecular mechanisms that instruct the patterning of entire organisms (the sequencing and imaging data are deposited in publicly available repositories and can be explored at http://digitalembryo.org). Our results demonstrate that the unbiased mapping of scRNA-seq data by the MorphoSeq framework yields a spatiotemporally resolved atlas of gene expression at the single-cell level in a developing embryo and links it to morphological features.

## Results

### Developmental Transcriptome of *P. mammillata*

**Figure S1:**
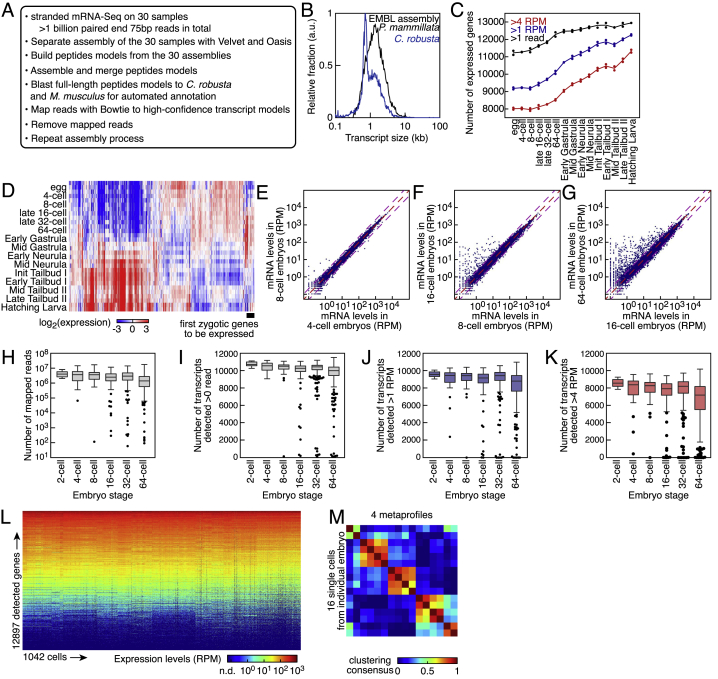
*P. mammillata* Developmental Transcriptome, Related to Figure 1 (A) Workflow for the *de novo* assembly of *P. mammillata* transcriptome. *Ciona robusta* was formerly known as *Ciona intestinalis*. (B) Distribution of *P. mammillata* transcript lengths. As comparison, the distribution for *C. robusta* (formerly known as *C. intestinalis*) is shown in blue. (C) Number of detected genes during development. Threshold in normalized reads (RPM: reads per million mapped reads): 1 (blue) and 4 (red) RPM. Black line: threshold at one mapped read. Staging was performed according to Hotta et al. (2007). (D) Hierarchical clustering of mRNA expression during *P. mammillata* embryogenesis. Staging was performed according to Hotta et al. (2007). (E) Expression profiles in 4- and 8-cell stage embryos as measured by RNA-Seq. Pink dashed lines indicate 2-fold expression changes. (F) Expression profiles in 4- and 16-cell stage embryos as measured by RNA-Seq. Pink dashed lines indicate 2-fold expression changes. (G) Expression profiles in 16- and 64-cell stage embryos as measured by RNA-Seq. Pink dashed lines indicate 2-fold expression changes. (H) Number of mapped reads for the 1084 single cells pooled by developmental stage. (I) Number of transcripts detected with at least one read across the 1042 single-cell transcriptomes pooled by developmental stage (RPM: reads per million mapped reads). (J) Number of transcripts with expression exceeding 1 read per million (RPM) across the 1042 single-cell transcriptomes pooled by developmental stage. (K) Number of transcripts with expression exceeding 4 reads per million (RPM) across the 1042 single-cell transcriptomes pooled by developmental stage. (L) Expression levels of 12,897 detected genes across 1042 single cells. Genes were sorted according to the median expression level across all cells. (M) Non-negative matrix factorization of gene expression profiles of the 16 single cells forming an individual 16-cell stage embryo. 4 metaprofiles split the embryo into the same 5 groups of 4, 4, 2, 4 and 2 cells with degraded consensus clustering compared to three metaprofiles.

Due to the lack of an annotated published *P. mammillata* transcriptome, we first set out to generate a high-quality *de novo* transcriptome assembly comprising all mRNAs expressed during embryonic development by sampling 15 developmental stages ranging from unfertilized eggs to hatching larvae. To circumvent the high degree of genomic polymorphism present in ascidians (Dehal et al., 2002), we devised a strategy of consensus building in the peptide space to consolidate 30 different assemblies into 12,945 gene models (Figures S1A and S1B; STAR Methods). We then analyzed the temporal gene expression changes at the whole-embryo level (Figures S1C and S1D). Only a handful of genes started to be expressed at the 8-cell stage (Figure S1E), increasing to tens of genes activated at the 16-cell stage (Figure S1F). As expected, the number of expressed genes increased during further development (Figures S1G and S1C).

### High-Resolution scRNA-Seq Captures the Bilateral Symmetry of the Embryo

Aiming to precisely resolve the spatial and temporal complexity of gene regulation during embryonic development, we thus turned to scRNA-seq. We collected single cells from individual embryos dissociated at 2- to 64-cell stages (Table S1) and, importantly, kept track of the embryo they belonged to. Due the very high fraction of cells profiled per individual embryo (100% up to the 16-cell stage and up to 90% for 32- and 64-cell stages), we can thus assess the degree of cell-to-cell variability both within a single embryo and between individuals (Figure 1C). The bilateral symmetry of *P. mammillata* provided an ideal internal control: in principle, symmetric pairs of cells from the left and right sides of the embryo should have highly similar expression profiles. In total, 1,042 cells from 58 embryos were analyzed at high depth, generating 6.65 billion reads and quantifying the expression of 8,542 ± 272 genes in individual cells (Figures S1H–S1L), including orthologs for 100% of the annotated *Ciona* transcription factors, zinc finger proteins, and signaling ligands expressed during embryogenesis (Imai et al., 2004). Overall, the resolution of the scRNA-seq was comparable to bulk mRNA-seq results confirming its very high depth.

At the 4-cell stage, two symmetric pairs of cells could already be distinguished in all embryos (Figures 1D–1F), with a set of 27 maternal mRNAs being asymmetrically segregated between the two cell types (Figure 1G). We leveraged the ability of non-negative matrix factorization (NMF) to cluster samples in an unsupervised way (Lee and Seung, 1999) to single out in a 16-cell embryo 5 groups of cells that were either pairs or consisted of an even number of cells (Figures 1H and S1M). These results suggested that our transcriptome resolution adequately captured the bilateral symmetry of the embryo. We challenged this notion at the more complex 64-cell stage where major lineage decision events have taken place (Imai et al., 2006). Using a 64-cell embryo with transcriptome information for 90% of cells, we could identify 7 symmetric cell pairs and 6 clusters formed by 4 or more cells (Figures 1I–1K).

### Cell Type Determination without Prior Knowledge

**Figure S2:**
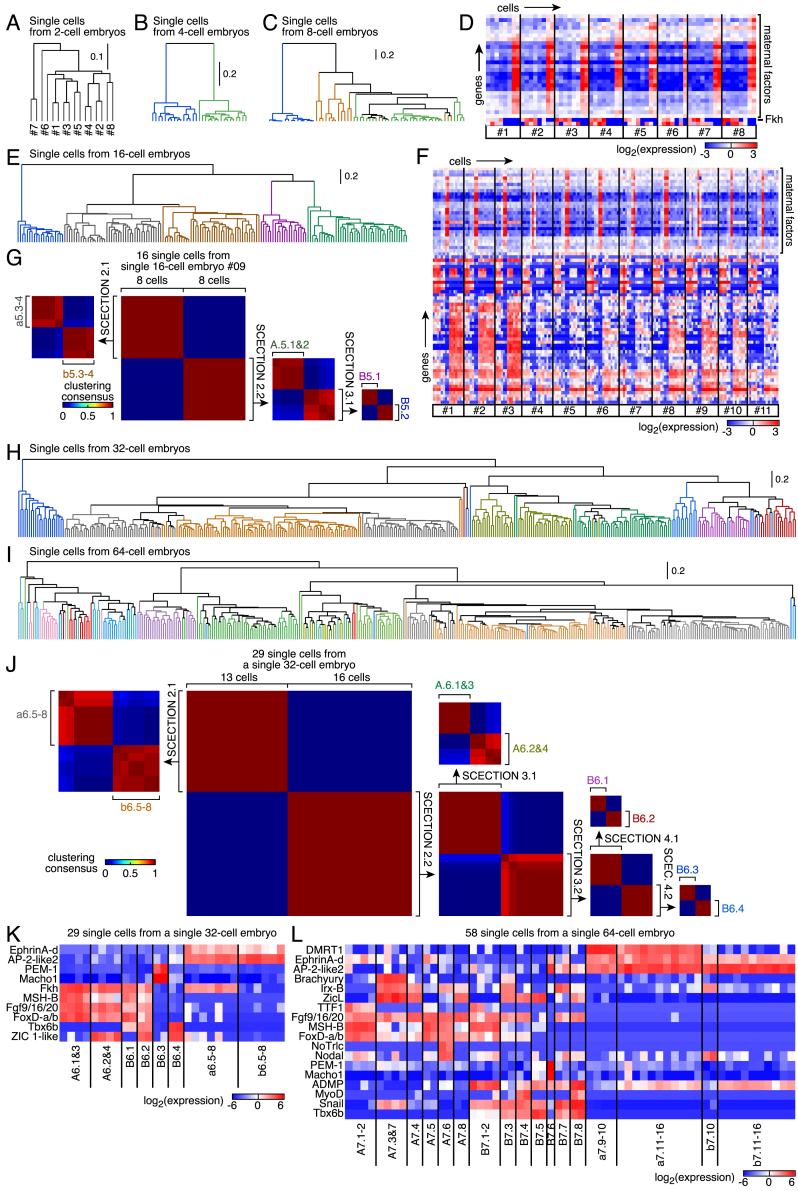
Classification of Single-Cell Gene Expression Profiles, Related to Figure 2 (A) Hierarchical clustering of single-cell transcriptome profiles of 2-cell embryos (embryo numbers are indicated). (B) Hierarchical clustering of single-cell transcriptome profiles of 4-cell embryos (branches are colored according to the cell type). (C) Hierarchical clustering of single-cell transcriptome profiles of 8-cell embryos (branches are colored according to the cell type). (D) Expression levels (as measured by scRNA-Seq) of genes that classify single cells of 8-cell embryos into three cell types. (E) Hierarchical clustering of single-cell transcriptome profiles of 16-cell embryos (branches are colored according to the cell type). (F) Expression levels (as measured by scRNA-Seq) of genes that classify five cell types in single cells of 16-cell embryos. (G) SCECTION identifies five cell types in 16-cell embryos.The groups of two cells that inherit the maternal factors belong to the germ cell lineage and are transcriptionally silent at that stage. (H) Hierarchical clustering of single-cell transcriptome profiles of 32-cell embryos (branches are colored according to the cell type determined by SCECTION). (I) Hierarchical clustering of single-cell transcriptome profiles of 64-cell embryos (branches are colored according to the cell type determined by SCECTION). (J) SCECTION applied to 29 single cells from an individual 32-cell embryo. SCECTION identified 8 cell types (SCEC.: SCECTION). (K) Expression levels (as measured by scRNA-Seq) of genes that classify 8 cell types in 29 single cells of a single 32-cell embryo. (L) Expression levels (as measured by scRNA-Seq) of genes that classify 17 cell types in 58 single cells of a single 64-cell embryo.

**Figure 2:**
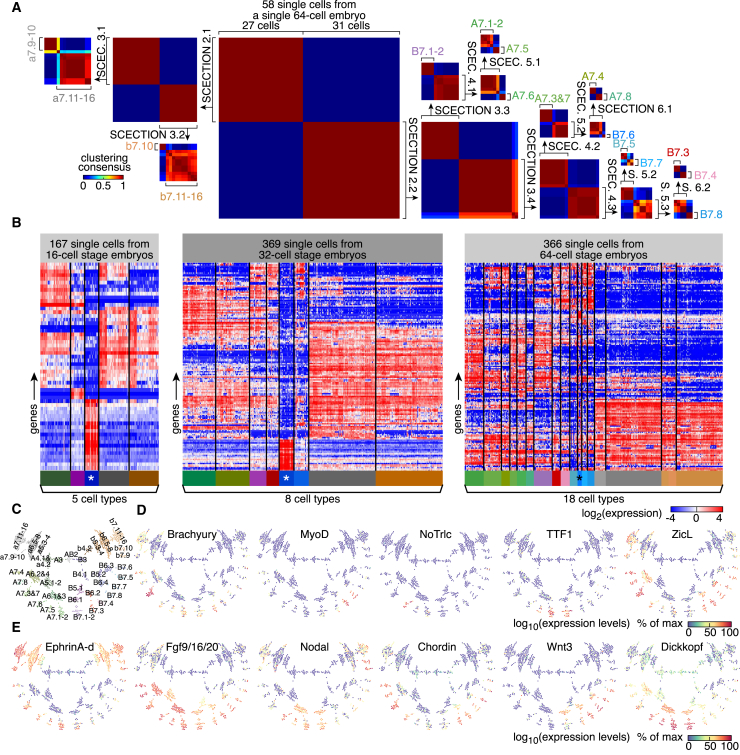
Cell Type Determination (A) Decomposition into cell types of 58 single cells from an individual 64-cell embryo by single-cell expression classification through iterations of NMF (SCECTION) (SCEC. and S., SCECTION). The clustering consensus of subset of cells obtained after each round of SCECTION is plotted. (B) Cell types identified in 16-, 32-, and 64-cell embryos (^∗^, germ cell lineage). (C) Two-dimensional representation of the different cell types. (D and E) Expression levels of transcription factors (D) and signaling pathway components (E). See also Figure S2.

We proceeded to stratify embryos into different cell types. At the 2-cell stage, individual single-cell gene expression profiles clustered by embryo (Figure S2A), whereas two cell types were apparent in 4-cell embryos (Figure S2B). At the 8-cell stage, 3 cell types could be distinguished in all embryos with the further asymmetric segregation of maternal mRNAs (Sardet et al., 2005) in a single cell pair (Figures S2C and S2D). Five cell types were singled out either by clustering expression profiles or by NMF in individual 16-cell embryos (Figures 1H, S2E, and S2F). Aiming to generalize cell type classification to more complex embryos, we designed the framework “single-cell expression classification through iterations of NMF” (SCECTION). Cells from a given embryo were iteratively split in subsets until minimal variation in gene expression remained within the subset (STAR Methods; Figure S2G). Subsets from different embryos at the same stage were then merged to ensure complete cell coverage and consistency across embryos, allowing us to define unique cell types. SCECTION could divide the 58 cells of a 64-cell embryo into 17 categories, including 10 symmetric cell pairs and 4 groups of 4 cells (Figure 2A). Although cells of 32- and 64-cell embryos were not readily clustered (Figures S2H and S2I), SCECTION identified 5, 8, and 18 different signatures at the 16-, 32-, and 64-cell stages, respectively (Figures 2B and S2J–S2L). This indicated a striking degree of diversification of single-cell gene expression profiles from the 16-cell stage onward. For each stage, we could derive a canonical embryo by integrating scRNA-seq data from multiple individual stage-matched embryos. Among the genes exhibiting cell-type-specific expression profiles were transcription factors and signaling ligands (Figures 2C–2E). In conclusion, our dataset allowed us to unbiasedly identify 18 different cell fates in 64-cell stage embryos.

### Temporal Mapping of scRNA-Seq Expression Profiles

**Figure 3:**
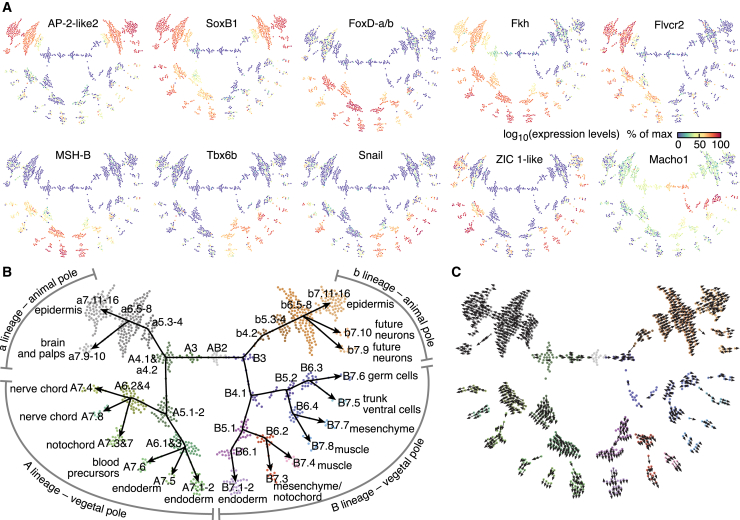
Single-Cell Gene Expression Profiles Reconstruct the Lineage Tree of the Embryo (A) Expression levels of marker genes. (B) Reconstructed lineage tree from scRNA-seq data. Development proceeds outward. Cell fates at the 64-cell stage are indicated. (C) RNA velocity field projected on the reconstructed lineage tree. Arrows show the local velocity of individual cells with measurable intron counts (STAR Methods). See also Figure S3.

**Figure S3:**
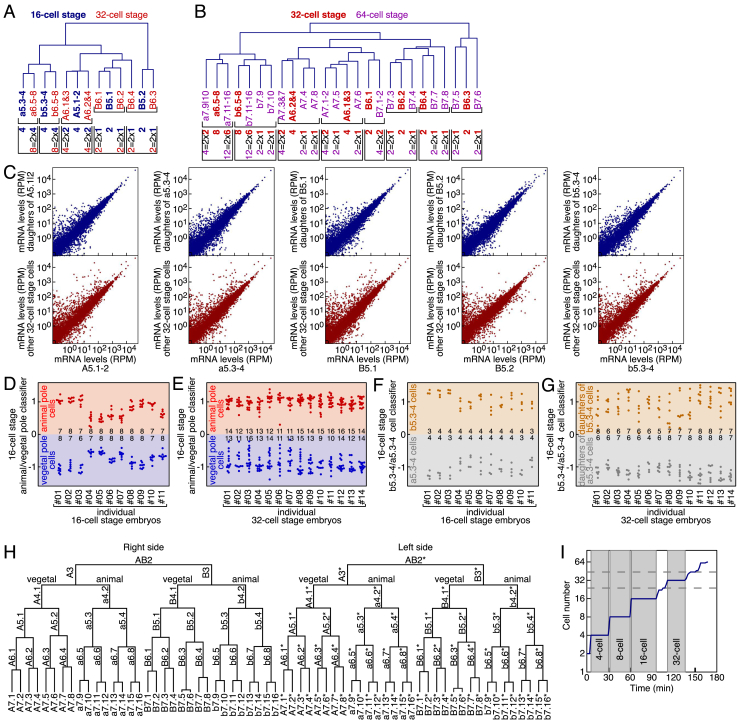
Temporal Mapping of scRNA-Seq Data, Related to Figure 3 (A) Average linkage hierarchical clustering of gene expression profiles of the 5 cell types at 16-cell stage (blue: A5.1-2, B5.1, B5.2, a5.3-4, b5.3-4) and the 8 cell types at 32-cell stage (red: A6.1&3, A6.2&4, B6.1, B6.2, B6.3, B6.4, a6.5-8, b6.5-8) determined by scRNA-Seq. Actual mother-daughter relationships are indicated by brackets under the cell type names. Number of cells belonging to each cell type in canonical embryos are indicated under the cell type names. (B) Average linkage hierarchical clustering of gene expression profiles of the 8 cell types at 32-cell stage (red: A6.1&3, A6.2&4, B6.1, B6.2, B6.3, B6.4, a6.5-8, b6.5-8) and the 18 cell types at 64-cell stage (purple: A7.1-2, A7.3&7, A7.4, A7.5, A7.6, A7.8, B7.1, B7.2, B7.3, B7.4, B7.5, B7.6, B7.7, B7.8, a7.9-10, a7.11-16, b7.9, b7.10, b7.11-16) determined by scRNA-Seq. Actual mother-daughter relationships are indicated by brackets underneath the cell type names. Number of cells belonging to each cell type in canonical embryos are indicated underneath the cell type names. (C) Comparison between expression levels of the five mother cell types at 16-cell stage and their respective daughter cell types at 32-cell stage (blue) or the other cell types (red) found in 32-cell embryos. (D, E) Projection of single-cell gene expression profiles of individual 16- (D) and 32-cell (E) embryos on the classifier that distinguishes vegetal pole cells form animal pole cells at the 16-cell stage. (F, G) Projection of gene expression profiles of single animal pole cells of individual 16- (F) and 32-cell (G) embryos on the classifier that distinguishes the two animal pole cell types at the 16-cell stage. (H) Lineage of *P. mammillata* up to the 64-cell stage. (I) Temporal evolution of the number of cells in a *P. mammillata* embryo up to the 64-cell stage. Horizontal dashed lines indicate 24 and 44 cells.

Due to the stereotypic nature of ascidian development, we hypothesized that the global gene expression profile of every cell might contain enough information to infer its lineage history in an unbiased manner. Possessing scRNA-seq data for every round of cell division allowed us to investigate the relatedness of gene expression profiles between mother and daughter cells. Some genes, among them the transcription factors AP-2-like2, SoxB1, FoxD-a/b, Fkh, MSH-B, Tbx6b, and Snail, increased their expression in specific cell types as development progressed (Figure 3A). Because asymmetric mRNA segregation occurred only in germ cells, we hypothesized that transcriptomes of mother and daughter cells should, in general, have a high degree of similarity unless cell fate specification occurs, which should involve the repression of pluripotency or the activation of lineage-specific genes in one or both daughter cells. We could match cell types unambiguously with one or more cell types at the next division (Figures S3A and S3B). This implied that daughter cells inherited the gene expression makeup of their mothers (Figures S3C–S3G), and we could distinguish specific sister cells by the differential expression of key genes. By iteratively matching all daughter cells with their respective mothers, we were able to delineate a complete lineage tree of the cell types identified by scRNA-seq (Figure 3B). This lineage was fully compatible with the actual developmental history of the corresponding embryonic cells (Figures S3H and S3I). A key assumption in our reconstruction was the continuity of gene expression between mothers and daughters. RNA velocity analysis (La Manno et al., 2018) supported the direction of the reconstructed trajectories, i.e., an increase in the expression of marker genes as development progressed (Figure 3C). The only notable exception was the germ cell lineage, which was transcriptionally silent up to the 64-cell stage. Therefore, cell fate specification proceeded mainly through the stepwise upregulation of specific gene sets that then maintained their expression at subsequent developmental stages.

### Spatial-Temporal Quantification of Early Development by 4D Imaging

**Figure S4:**
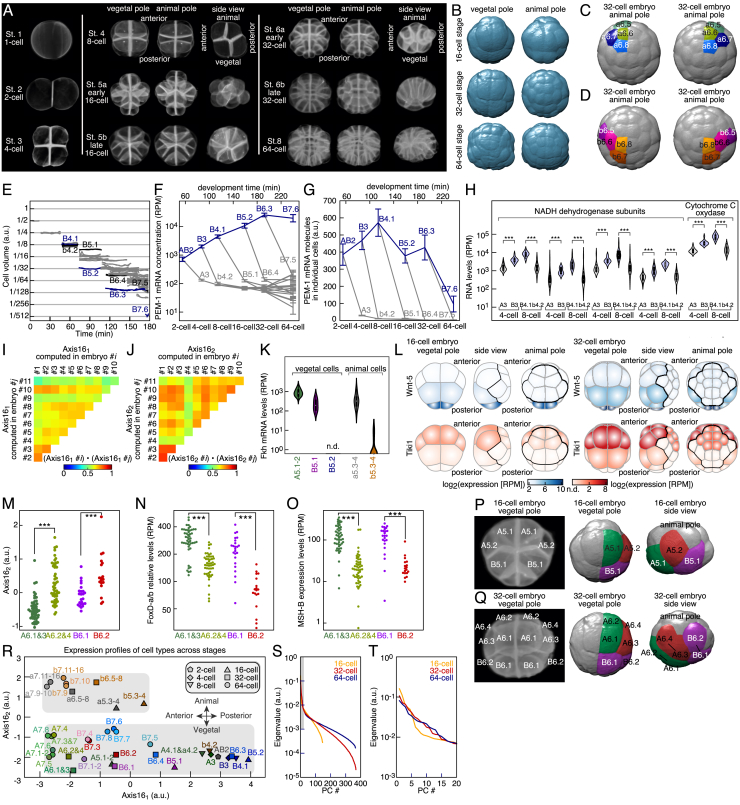
Early Development of *P. mammillata*, Asymmetric RNA Inheritance and Spatial Mapping of scRNA-Seq Data, Related to Figure 4 (A) Live imaging of *P. mammillata* early development. Images of embryos expressing membrane-bound Citrine were rendered using Chimera (Pettersen et al., 2004). (B) Morphology of *P. mammillata* embryos. Segmented membrane signal of the vegetal and animal poles at 16-, 32 and 64-cell stages. (C) Progeny of the two a4.2 cells (one for each side of the embryo) after two rounds of division at the 32-cell stage forming two pairs of sister cells: a6.5 and a6.6 (green) and a6.7 and a6.8 (blue). (D) Progeny of the two b4.2 cells (one for each side of the embryo) after two rounds of division at the 32-cell stage forming two pairs of sister cells: b6.5 and b6.6 (purple) and b6.7 and b6.8 (orange/red). (E) Temporal evolution of the cellular volumes as measured by light sheet microscopy. Blue: germ cell lineage (B4.1, B5.2, B6.3, B7.6); black: corresponding sister cells (b4.2, B5.1, B6.4, B7.5 respectively; gray: other cells. (F) Expression levels of the maternal factor PEM-1 as measured by scRNA-Seq. Blue line: germ cell lineage (giving rise to cell B7.6 at 64-cell stage). Gray lines: all the other lineages. Data represented as mean ± SEM. (G) Total mRNA levels of the maternal factor PEM-1 as measured by scRNA-Seq and scaled by cellular volume measured by light sheet microscopy. Blue line: germ cell lineage (giving rise to cell B7.6 at 64-cell stage). Gray lines: all the other lineages. Data represented as mean ± SEM. (H) Violin plots of expression levels for five mitochondrial transcripts coding for four subunits of the NADH dehydrogenase and cytochrome C oxydase. ^∗∗∗^: p < 0.001, Kruskal-Wallis test. (I, J) Comparison of the eleven different Axis16_1_ (I) and Axis16_2_ (J) determined independently for the eleven 16-cell embryos assessed. p < 10^−32^ for Axis16_1_ and p < 10^−46^ for Axis16_2_ to get the reported degree of colinearity between two random vectors in the 595 dimensions used to compute the principal component analysis. (K) Violin plot of the forkhead transcription factor Fkh expression levels at 16-cell stage (n.d.: not detected). (L) Expression levels of Wnt-5 (blue) and the metalloprotease Tiki1 (red) at the 16-cell stage and the 32-cell stage as measured by scRNA-Seq (n.d: not detected). (M) Projection on Axis16_2_ of single-cell transcriptomes of 47 A6.1&3, 48 A6.2&4, 24 B6.1 and 18 B6.2 cells (Comparison between A6.1&3 and A6.2&4 cells: p = 5 × 10^−11^; Comparison between B6.1 and B6.2 cells: p = 10^−4^, Kruskal-Wallis test). (N) FoxD-a/b expression levels in 47 A6.1&3, 48 A6.2&4, 24 B6.1 and 18 B6.2 cells (Comparison between A6.1&3 and A6.2&4 single cells: p = 7 × 10^−11^; Comparison between B6.1 and B6.2 cells: p = 10^−6^, Kruskal-Wallis test). (O) MSH-B expression levels in 47 A6.17&3, 48 A6.2&4, 24 B6.1 and 18 B6.2 cells (Comparison between A6.1&3 and A6.2&4 single cells: p = 5 × 10^−13^; Comparison between B6.1 and B6.2 cells: p = 4 × 10^−6^, Kruskal-Wallis test). (P, Q) Membrane signal and segmented membrane signal of the vegetal pole at 16- (P) and 32-cell stages (Q). A6.2, A6.4 and B6.2 lie closer to the animal pole compared to their respective sister cells A6.1, A6.3 and B6.1. Sister cells are linked by bars and have the same color as their mother. (R) Projection on Axis16_1_ and Axis16_2_ of average expression profiles for all cell types identified. (S, T) Eigenvalue spectrum of the principal components (PC) computed with scRNA-Seq expression profiles of 16-, 32- or 64-cell embryos (yellow, red and blue respectively). Full spectrum (S) or zoom on the first 20 PCs (T, corresponding to the gray shaded area in S) are shown.

To precisely capture spatial cell dynamics in the developing embryo, we performed *in toto* high-resolution imaging of *P. mammillata* embryonic development from the 2- to 64-cell stage by using multi-view light sheet microscopy (Krzic et al., 2012). Selective plane illumination microscopy (SPIM) videos of embryos expressing fluorescent nucleus- and membrane-targeted probes were segmented, and individual cells were tracked (Figures S4A and S4B; Videos S1, S2, and S3; STAR Methods). The quantification of our 4D imaging data yielded a complete digital representation of embryonic development up to gastrulation, quantifying the temporal evolution of cell positions and volumes, membrane shapes, and cell-cell neighbor contacts with a 2-min temporal resolution. Owing to the lack of cell migration in the early embryo, the spatial position of each cell was closely correlated with its lineage history, such that cells arising from sister lineages remained in spatial proximity (Figures S4C and S4D). We noticed a very strong asymmetric volume partitioning in one lineage from the 16-cell stage, yielding the future germ cells that were 8-fold smaller than the average cell at the 64-cell stage (Figure S4E). The enrichment of the 27 maternal factors relative to other genes in the germ cell lineage (Figure S4F; STAR Methods) allowed us to infer that cell divisions in that lineage gave sister cells of unequal volume, a prediction validated by the imaging data. Moreover, the total mRNA numbers of the maternal factors were constant up to the 64-cell stage (Figure S4G), which is consistent with our bulk mRNA-seq data. Strikingly, this was the sole example of asymmetric segregation of mRNAs during cell division in our dataset. Interestingly, germ cell specification was also accompanied by an enrichment of mitochondrial transcripts (Figure S4H), consistent with the known asymmetric partitioning of mitochondria into this lineage (Zalokar and Sardet, 1984).

**Figure 4:**
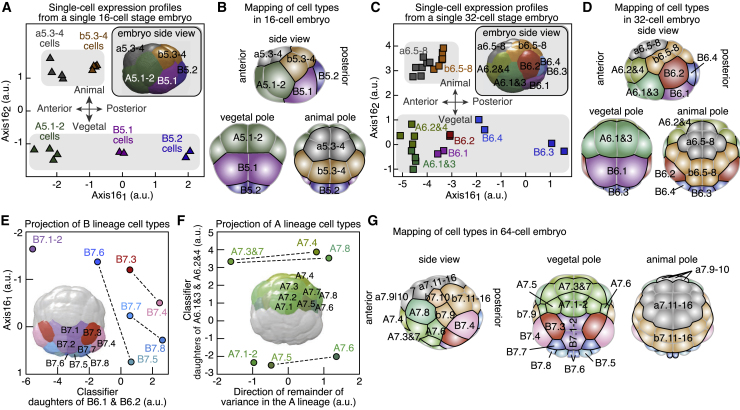
Single-Cell Gene Expression Profiles Map the Spatial Organization of the Embryo (A) Projection on Axis16_1_ and Axis16_2_ of single-cell transcriptomes of all 16 cells of a 16-cell embryo. Inset: 16-cell embryo side view. (B) Mapping of cell types in a 16-cell embryo. (C) Projection on Axis16_1_ and Axis16_2_ of single-cell transcriptomes of 29 cells of a 32-cell embryo. Inset: 32-cell embryo side view. (D) Mapping of cell types in a 32-cell embryo. (E and F) (E) or of A lineage cell types on the classifier of the daughters of A6.1 and 3 and A6.2 and 4 and the direction of the remainder of the variance in the A lineage. (F). Inset: 64-cell embryo vegetal pole. B7.6 belongs to the germ cell lineage. Dashed lines link sister cell types. (G) Mapping of cell types in a 64-cell embryo. See also Figures S4 and S5 and Table S2.

Video S1. Live Imaging of *P. mammillata* development by Using Light Sheet Microscopy, Related to Figure 4

Video S2. Live Imaging of *P. mammillata* Development by Using Light Sheet Microscopy, Related to Figure 4

Video S3. Segmentation and Tracking of *P. mammillata* Development, Related to Figure 4

### Spatial Mapping of scRNA-Seq Expression Profiles

Next, we set out to map single-cell gene expression profiles onto their spatial position in the embryo at each developmental stage. Performing this analysis for all stages would yield a spatiotemporally resolved transcriptome atlas at the single-cell level. We reasoned that the local signaling environment of individual cells might impact their gene expression and, therefore, enable us to determine their relative positions in the embryo. We first focused on the 16-cell stage when all cells (except B5.2 cells, which will give rise to the future germ cells) were transcriptionally active. To recover the spatial coordinates of the cells, we devised a method relying on principal-component analysis to identify the axes that best discriminate the cell types of the embryo. At the 16-cell stage, this method defined two axes, namely, Axis16_1_ and Axis16_2_. The projection on these two axes of the expression profiles of all 16 cells of one 16-cell embryo separated the 5 cell types determined by SCECTION (Figure 4A). Axis16_1_ mapped the anterior-posterior axis of the embryo, with the posterior position of the germ cells defining its orientation. Moreover, Axis16_2_ split the embryo in two groups of eight cells, faithfully distinguishing the animal and vegetal poles of the embryo. Notably, the relative positions of the projections of single-cell expression profiles in the Axis16 space were strikingly similar to the corresponding cell’s physical positions within the 16-cell embryo (Figures 4A and 4B). These spatial axes were reproducibly recovered for each of the 11 analyzed individual 16-cell stage embryos (Figures S4I and S4J). Among the top genes contributing to Axis16_1_ (antero-posterior axis), the transcription factor Fkh had graded expression from anterior to posterior (Figure S4K). Interestingly, Wnt-5 and the Wnt-degrading metalloprotease Tiki1 (Zhang et al., 2012) formed opposite gradients along the antero-posterior axis (Figure S4L). Indeed, Wnt is critical for antero-posterior axis specification (Petersen and Reddien, 2009). The posterior B5.2 cells inherit maternal factors that establish cellular asymmetry during early development (Negishi et al., 2007), among them Wnt-5 and the metalloprotease Tolloid. Overall, the combination of scRNA-seq and imaging data allowed the mapping of single-cell gene expression profiles onto their respective spatial locations at the 16-cell stage.

The same map might be used to determine the relative position of daughter cell types at the more complex 32-cell stage. The projections of single-cell transcriptomes of a 32-cell embryo onto the Axis16 space defined by 16-cell stage gene expression resulted in the separation of cells into the SCECTION-identified cell types and also faithfully recapitulated the spatial organization of the embryo (Figures 4C and 4D). Notably, Axis16_2_, defined at 16-cell stage, separated daughters of A5.1, A5.2, and B5.1 into the respective cell clusters at the 32-cell stage, consistent with the SCECTION results (Figure S4M). A closer inspection of expression differences between sister pairs showed that A6.2, A6.4, and B6.2 cells had lower levels of vegetal-pole-specific transcription factors than their sister cells (Figures S4N and S4O). This finding is consistent with the origin of these sister cells from the orientation of the cell division axis that pushed these cells toward the animal pole (Figures S4P and S4Q).

As the expression of signaling ligands dynamically changed during embryogenesis (Figure 2E), it stands to reason that the axes determined at an earlier stage will no longer be able to discriminate sister cell types as development progresses. Indeed, projecting all cell types onto the Axis16 space did not resolve the relative positions of the vegetal pole cell types at the 64-cell stage (Figure S4R). However, new axes defined using the variance in gene expression at the 32- and 64-cell stages in the A and B lineages enabled us to map these cell types (Figures 4E and 4F). Although one axis matched the antero-posterior axis, the other axis was along the left-right axis of the vegetal pole. Despite a large number of identified cell types, the dimensionality of the data was rather small (Figures S4S and S4T). In summary, each cell type could be mapped onto its physical position in the embryo for each developmental stage (Figures 4B, 4D, and 4G).

**Figure S5:**
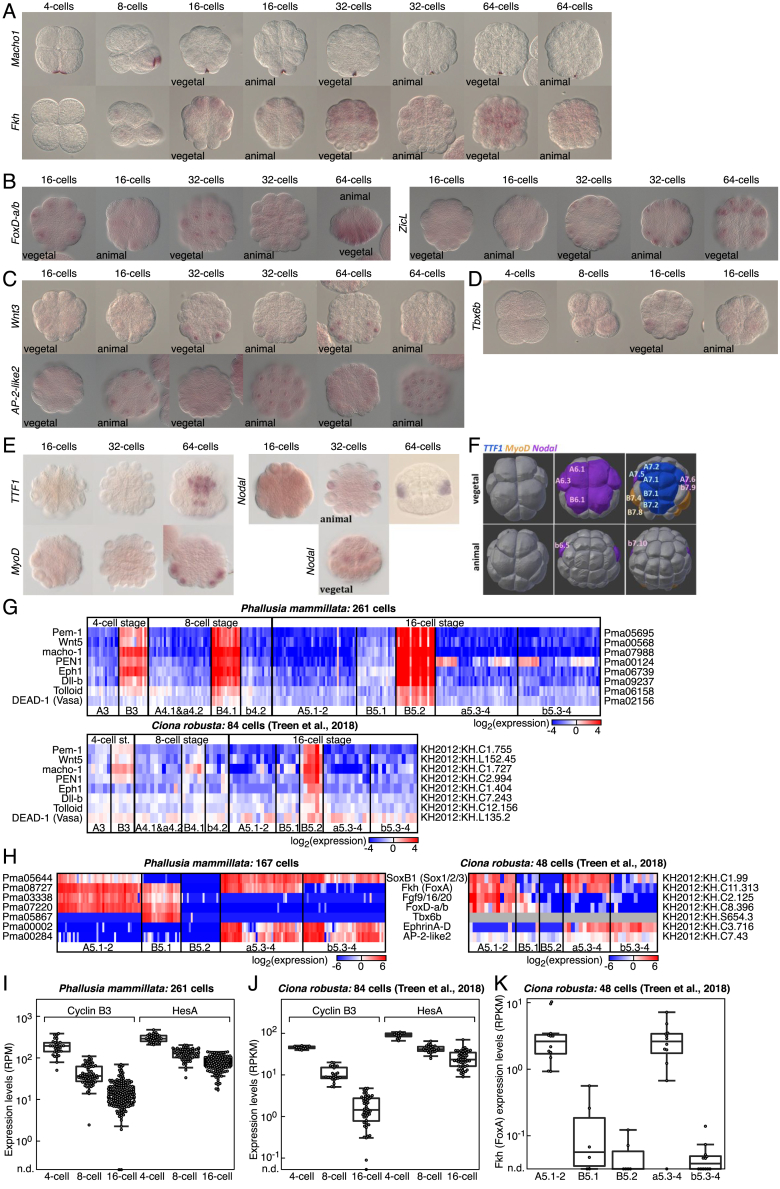
Validation of the Spatial Mapping of Single-Cell Gene Expression Profiles, Related to Figure 4 (A) Whole mount *in situ* hybridization of expression profiles of *Macho1* and *Fkh* at 4- to 64-cells stages. (B) Whole mount *in situ* hybridization of expression profiles of *FoxD-a/b* and *ZicL* at 16- to 64-cells stages. (C) Whole mount *in situ* hybridization of expression profiles of *Wnt3* and *AP-2-like2* at 16- to 64-cells stages. (D) Whole mount *in situ* hybridization of expression profiles of *Tbx6b* at 4- to 16-cells stages. (E) Whole mount *in situ* hybridization of expression profiles of *TTF1*, *MyoD* and *Nodal* at late 16-cells, late 32-cells and 64-cells stages (vegetal poles are shown unless indicated. (F) Cell identity of cells expressing *TTF1*, *MyoD* and *Nodal* represented in segmented embryos. Vegetal and animal indicate the hemisphere view of the embryo. (G) Expression levels of maternal factors asymmetrically inherited in the germ cell lineage (cells B3, B4.1 and B5.2) as measured by scRNA-Seq in *Phallusia mammillata* and *Ciona robusta* (*Ciona robusta* data is from Treen et al., 2018). All these factors are known to be asymmetrically partitioned from 4-cell stage onward (and 16-cell stage for DEAD-1) in *Ciona robusta* (Sardet et al., 2005). *Phallusia* gene IDs are identical to the ones from http://digitalembryo.org. (H) Expression levels of marker genes in 16-cell stage cells as measured by scRNA-Seq in *Phallusia mammillata* and *Ciona robusta* (*Ciona robusta* data is from Treen et al., 2018). Tbx6b was not detected in *Ciona robusta* by scRNA-Seq by Treen et al. (2018) although Treen et al. (2018) could detect it by qPCR in embryos and by whole mount *in situ* hybridization in B5.1 cells. *Phallusia* gene IDs are identical to the ones from http://digitalembryo.org. (I, J) Expression levels of CyclinB3 and HesA in 4- to 16-cell stage cells as measured by scRNA-Seq in *Phallusia mammillata* (I) and *Ciona robusta* (J) (*Ciona robusta* data is from Treen et al., 2018, RPM: reads per million mapped reads, RPKM: reads per kilo base per million, n.d.: not detected).(K) Expression levels of the forkhead transcription factor Fkh (FoxA) in 16-cell stage cells of *Ciona robusta* (*Ciona robusta* data is from Treen et al., 2018, n.d.: not detected).

We then validated the expression territories of key classifier genes by using whole-mount *in situ* hybridization (ISH) (Figures S5A–S5F). Moreover, our expression territories were fully compatible with reported expression patterns both in *Phallusia* (Madgwick et al., 2019) or *Ciona* (Imai et al., 2004). Notably, comparing our data with scRNA-seq data in *Ciona* by Treen et al. (2018), we found that there was conservation of the asymmetric division of maternal factors in the germ cell lineage (Figure S5G), the expression of marker genes (Figure S5H), Cyclin B3 (Figures S5I and S5J), and Fkh (Figure S5K). All these observations were in accordance with the conservation of expression patterns in ascidians (Wada et al., 1998).

Thus, the single-cell expression profiles contained intrinsic information that allowed us to objectively deduce their original spatial position within the embryo. The reconstruction of both spatial position and history of single-cell transcriptomes yielded a complete unbiased delineation of genome-wide expression trajectories for every single cell of the embryo up to gastrulation.

### Integration of Imaging and scRNA-Seq Data Identifies Patterned Expression of Cell Adhesion Molecules

We next integrated the spatiotemporal mapping of averaged single-cell transcriptomes from multiple embryos with the 4D lineage obtained by SPIM imaging to create a canonical “virtual embryo” that captures the interrelationship of gene expression, cell morphology, and lineage history for every single cell throughout development (http://digitalembryo.org). To intuitively demonstrate the power of this concept, we automatically generated 4D movies of virtual whole-mount ISHs to capture dynamic gene expression patterns at a single-cell resolution (see Video S4 for an example and http://digitalembryo.org for all 12,945 genes).

Video S4. Expression Patterns of Fgf6/19/20 between 4- and 64-Cell Stage, Related to Figure 4

**Figure 5:**
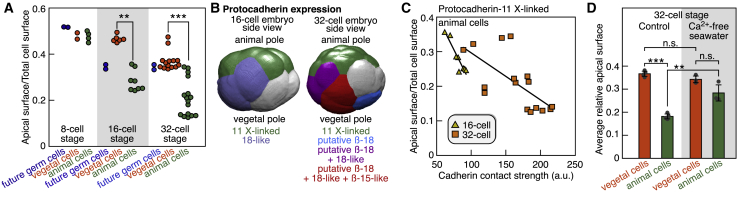
Integration of Imaging and scRNA-Seq Data Identify Patterned Protocadherin Expression (A) Relative apical surface of cells (p = 0.002 at 16-cell stage and p = 7.10^−6^ at 32-cell stage, Kruskal-Wallis test). (B) Expression territories of four protocadherins. (C) Comparison between the estimated protocadherin-11 X-linked contact strength and the relative apical surface in animal cells (r = −0.94 and p = 0.0006 at 16-cell stage, r = −0.67 and p = 0.004 at 32-cell stage, Pearson’s correlation coefficient). (D) Average relative apical surface of animal and vegetal cells of control embryos and embryos treated with Ca^2+^-free seawater at 32-cell stage (p = 1.5 10^−6^ for the animal and vegetal comparison in control embryos, n = 4; p = 0.18 for the animal and vegetal comparison in Ca^2+^-free-treated embryos, n = 3; p = 0.01 for the animal cells control/Ca^2+^-free comparison; p = 0.17 for the vegetal cells control/Ca^2+^-free comparison; n.s., non-significant; two-tailed t test; data represented as mean ± SD). See also Figure S6.

**Figure S6:**
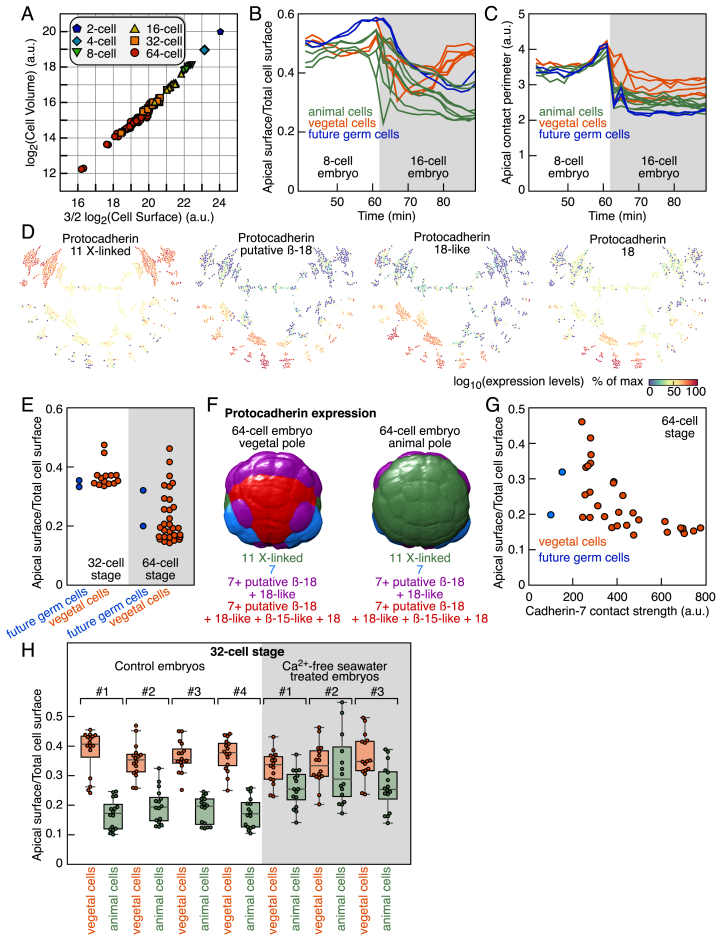
Morphological Changes and Patterned Expression of Cell Adhesion Molecules, Related to Figure 5 (A) Comparison between cell volume and surface as measured by SPIM imaging from 2-cell to 64-cell stages (r = 0.97 and p = 2.10^−76^, Pearson’s correlation coefficient). (B) Temporal evolution of the relative apical surface from 8-cell to 16-cell stage as measured by SPIM imaging. (C) Temporal evolution of the apical contact perimeter from 8-cell to 16-cell stage as measured by SPIM imaging. (D) Expression levels of four protocadherins in the different cell types. (E) Relative apical surface of vegetal cells at the 32- and 64-cell stages. (F) Expression territories of six cadherins and protocadherins at the 64-cell stage as measured by scRNA-Seq. Territories were displayed on rendered segmented membrane signal from SPIM imaging. (G) Comparison between the estimated cadherin-7 contact strength and the relative apical surface in animal cells at the 64-cell stage (r = −0.62 and p = 0.0001, Pearson’s correlation coefficient). (H) Relative apical surface of animal and vegetal cells at 32-cell stage of control embryos and embryos treated with Ca^2+^-free seawater.

Our quantification of 4D SPIM imaging data detected a decrease of the relative apical surface (i.e., the surface facing the environment) of animal cells compared to vegetal cells at the 16- and 32-cell stages (Figure 5A). Although cell volumes and cell surfaces were highly correlated (Figure S6A), the gradual decrease in the relative apical surface of animal cells occurred at a constant apical perimeter (Figures S6B and S6C). We turned to our scRNA-seq results for genes with differential expression between animal and vegetal cells that might explain these morphological changes. Interestingly, an analysis of cell adhesion molecules showed that the embryo became progressively stratified into distinct spatial territories through dynamic expression of different cadherin family proteins (Figures 5B and S6D). We defined a measure of the homophilic cadherin cell-cell contacts, the cadherin contact strength, as the product of the relative cadherin expression in neighboring cells and their apical contact length. The computed Protocadherin-11 X-linked contact strength was anti-correlated with the relative apical surface of animal cells at 16- and 32-cell stages (Figure 5C). Heterogeneity in the relative apical surface of vegetal cells occurred primarily at the 64-cell stage (Figure S6E). Cadherin-7 was upregulated in vegetal cells at the 64-cell stage (Figure S6F), and its computed contact strength was anti-correlated with the relative apical surface of vegetal cells (Figure S6G). Incubating 32-cell embryos in calcium-free seawater abolished the asymmetry in the relative apical surface between embryonic poles (Figures 5D and S6H), validating cadherin involvement in this process. Thus, both animal and vegetal poles had specific cadherin expression patterns that were correlated with changes in cellular apical surfaces. In the future, it would be interesting to connect our findings to other cell biological processes that influence cell shape. Altogether, this analysis exemplifies the strength of combining quantitative imaging data with scRNA-seq to uncover the links between morphology and gene expression.

### Variability of Gene Expression

**Figure S7:**
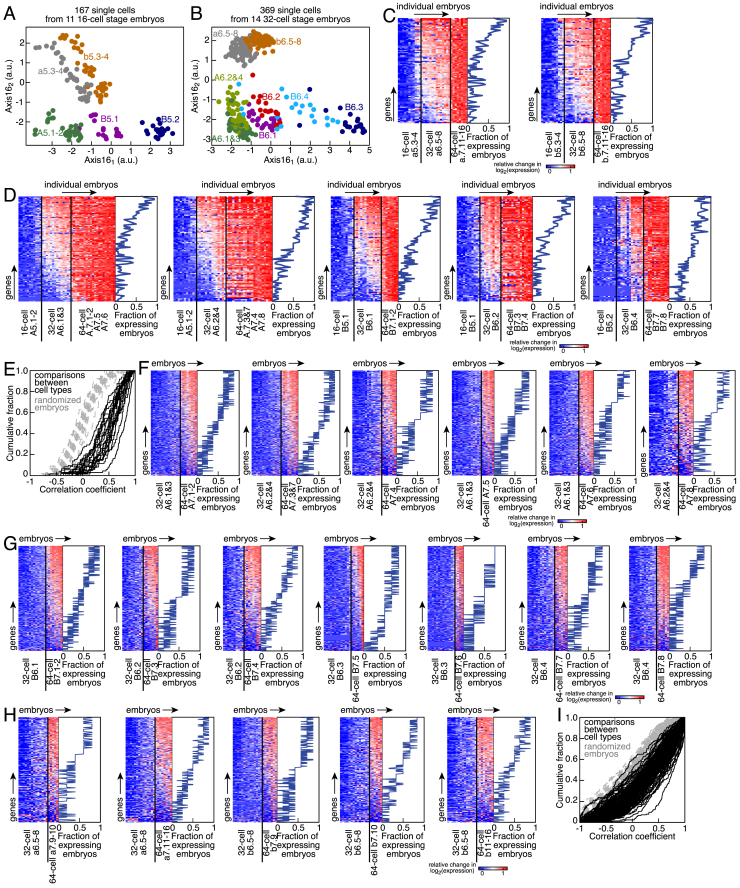
Variability of Gene Expression in *P. mammillata* Embryos, Related to Figure 6 (A) Projection on Axis16_1_ and Axis16_2_ of single-cell transcriptomes of eleven 16-cell embryos. Green: A5.1-2 cells; purple: B5.1 cells; blue: B5.2 cells; gray: a5.3-4 cells; orange: b5.3-4 cells. (B) Projection on Axis16_1_ and Axis16_2_ of single-cell transcriptomes of fourteen 32-cell embryos. Dark green: A6.1&3; light green: A6.2&4; purple: B6.1 cells; red: B6.2 cells; dark blue: B6.3 cells; light blue: B6.4 cells; gray: a6.5-8 cells; orange: b6.5-8 cells. (C) Relative changes in the log-transformed expression levels of genes that were upregulated in the animal pole cells a6.5-8 and b6.5-8, and in their respective mother and daughter cell types. Expression levels were averaged in individual embryos and embryos were sorted along the x axis according to the global upregulation of this set of genes, the same order of embryos was used for the seven heatmaps. The fraction of 32-cell embryos expressing each gene at a level greater than 0.5 × the log-transformed average expression level at the 64-cell stage is plotted for each cell type. (D) Relative changes in the log-transformed expression levels of genes that were upregulated in the vegetal pole cells A6.1&3, A6.2&4, B6.1, B6.2 and B6.4, and in their respective mother and daughter cell types. Expression levels were averaged in individual embryos and embryos were sorted along the x axis according to the global upregulation of this set of genes, the same order of embryos was used for the seven heatmaps. The fraction of 32-cell embryos expressing each gene at a level greater than 0.5 × the log-transformed average expression level at the 64-cell stage is plotted for each cell type. (E) Correlation coefficient of expression levels of genes that were upregulated in all the combination of two somatic cell types at 32-cell stage. Expression levels were averaged in individual embryos (solid black lines, dotted gray lines: randomized embryos). (F) Relative changes in the log-transformed expression levels of genes that were upregulated in the vegetal pole cells of the A lineage A7.1-2, A7.3&7, A7.4, A7.5, A7.6 and A7.8, and in their respective mother cell types. Expression levels were averaged in individual embryos and embryos were sorted along the x axis according to the global upregulation of this set of genes. The fraction of 64-cell embryos expressing each gene at a level greater than 0.5 × the log-transformed maximal expression level at the 64-cell stage is plotted for each cell type. (G) Relative changes in the log-transformed expression levels of genes that were upregulated in the vegetal pole cells of the B lineage B7.1-2, B7.3, B7.4, B7.5, B7.6, B7.7 and B7.8, and in their respective mother cell types. Expression levels were averaged in individual embryos and embryos were sorted along the x axis according to the global upregulation of this set of genes. The fraction of 64-cell embryos expressing each gene at a level greater than 0.5 × the log-transformed maximal expression level at the 64-cell stage is plotted for each cell type. (H) Relative changes in the log-transformed expression levels of genes that were upregulated in the animal pole cells a7.9-10, a7.11-16, b7.9, b7.10 and b7.11-16, and in their respective mother cell types. Expression levels were averaged in individual embryos and embryos were sorted along the x axis according to the global upregulation of this set of genes. The fraction of 64-cell embryos expressing each gene at a level greater than 0.5 × the log-transformed maximal expression level at the 64-cell stage is plotted for each cell type. (I) Correlation coefficient of expression levels of genes that were upregulated in all the combination of two somatic cell types at 64-cell stage. Expression levels were averaged in individual embryos (solid black lines, dotted gray lines: randomized embryos).

**Figure 6:**
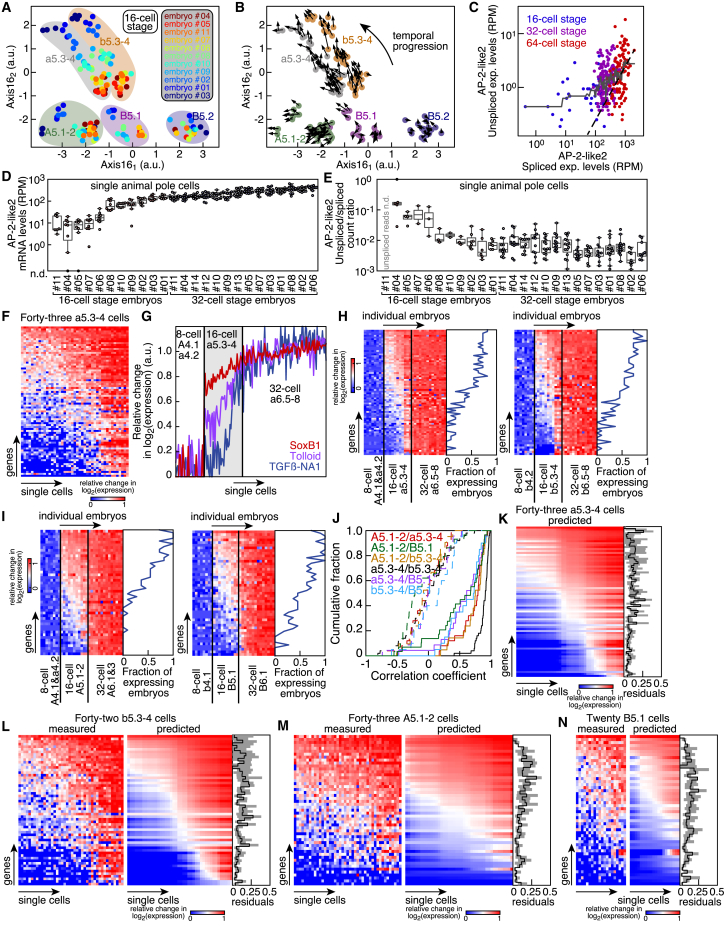
Temporal Variability in Gene Expression (A) Projection on Axis16_1_ and Axis16_2_ of single-cell transcriptomes of 11 16-cell embryos. Cell color according to embryo. (B) RNA velocity field projected on Axis16_1_ and Axis16_2_. Arrows show the local velocity of individual cells (with measurable intron counts) of 16-cell embryos. Cell color according to cell type. (C) Comparison between spliced and unspliced read counts of AP-2-like2 in single cells at the 16-, 32-, or 64-cell stage (blue, purple, and red respectively; gray, median filter; dashed black line, γ fit; STAR Methods). (D and E) Expression levels (D) and ratio of unspliced to spliced read counts (E) of AP-2-like2 in single animal pole cells of individual embryos (n.d., not detected). (F) Relative expression levels of upregulated genes in a5.3-4 cells. (G) Relative expression levels of SoxB1, Tolloid, and TGFβ-NA1 in a5.3-4 cells, their mother and daughter cell types. (H and I) Relative expression levels of genes upregulated in the animal (H) and vegetal somatic cell types (I) (excluding the germ cell lineage) of 16-cell stage embryos, their mother and daughter cell types. Levels were averaged in individual embryos. The same order of embryos was used for the 4 heatmaps. The fraction of 16-cell embryos expressing each gene at a level greater than 0.5 × the log-transformed average expression level at the 32-cell stage is indicated. (J) Correlation coefficient of expression levels of genes that were upregulated in all the combination of two somatic cell types at 16-cell stage (solid lines; dotted lines, randomized embryos). (K) Relative expression levels of upregulated genes in a5.3-4 cells predicted with a model of linear variation in time (STAR Methods). Residuals for individual genes are indicated (black line, median; gray shade, interquartile range). (L–N) Relative expression levels of upregulated genes as measured by scRNA-seq or predicted with a model of linear variation in time in b5.3-4 (L), A5.1-2 (M), and B5.1 (N) cells. Residuals for individual genes are indicated (black line, median; gray shade, interquartile range). See also Figure S7.

We next mined our dataset to study the extent of noise and variability in gene expression during development, both within single embryos and between individuals. We, thus, projected and overlaid the single-cell expression profiles from various 16- or 32-cell embryos on the Axis16 space (Figures S7A and S7B). Despite robust classification across all embryos, the expression profiles of individual cells exhibited large variation, which was particularly pronounced for the a5.3, a5.4, b5.3, and b5.4 cells and their progeny that give rise to the ectoderm. This spread was clearly a consequence of inter-embryo variability (Figure 6A). To explain this variation, we turned to RNA velocity analysis (La Manno et al., 2018) of individual cells of 16-cell embryos. The velocity field was aligned with the dispersion of animal pole cells in the Axis16 space (Figure 6B), indicating that the genes contributing to the axis were actively upregulated in individual cells.

Thus, this inter-embryo variability was most likely caused by minor differences in the temporal progression of embryonic development at the time of collection. There was an enrichment in unspliced read counts at low expression levels of the animal pole marker AP-2-like2, indicating an increase in expression (Figure 6C). Moreover, AP-2-like2 showed embryo-dependent states of upregulation, but its expression at the later 32-cell stage was not as variable (Figure 6D). There was a concomitant decrease in its relative intron counts, which plateaued at the 32-cell stage (Figure 6E).

Individual cells and individual embryos could upregulate genes in either a coordinated or an uncoordinated manner. Expression profiles of single a5.3-4 cells at the 16-cell stage showed that individual cells expressed progressively more genes and at higher levels (Figure 6F). Genes exhibited three trends of upregulation, namely, one class of genes in which all profiled cells expressed high levels, one class for which the expression levels of profiled cells spanned a larger spectrum, and finally a class of genes that were upregulated in only a small fraction of cells (Figure 6F). There was a general trend of ramping up expression levels across the upregulated genes once they had detectable expression in individual cells (Figures 6F and 6G). This suggested that genes were upregulated with different timing of activation in individual cells in a coordinated manner. To test whether this observation held true across cell types and embryos, we performed a similar analysis by averaging expression profiles for each cell type in individual embryos. We found that both the number of genes and their extent of upregulation increased in individual 16-cell embryos, with all these genes having an expression that plateaued in daughter cell types (Figures 6H and 6I). Moreover, genes that were upregulated in two different cell types were expressed in a highly correlated manner (Figure 6J). Similarly, genes were upregulated in a coordinated and temporally graded manner in all the somatic cell types at the 32-cell stage (Figures S7C–S7E) and in all the 18 cell types at the 64-cell stage (Figures S7F–S7I). In fact, relative changes in log-transformed gene expression levels in individual cells of 16-cell embryos were well approximated by a model of linear variation in time (Figures 6K–6N; STAR Methods), stressing that gene upregulation occurred in a coordinated, highly reproducible, and temporally ordered manner. Thus, profiling single cells from pooled and dissociated embryos (a strategy commonly used by previous studies) would introduce a major source of noise if there are minor differences in staging between individual embryos.

### Precision of Gene Expression Regulation

**Figure 7:**
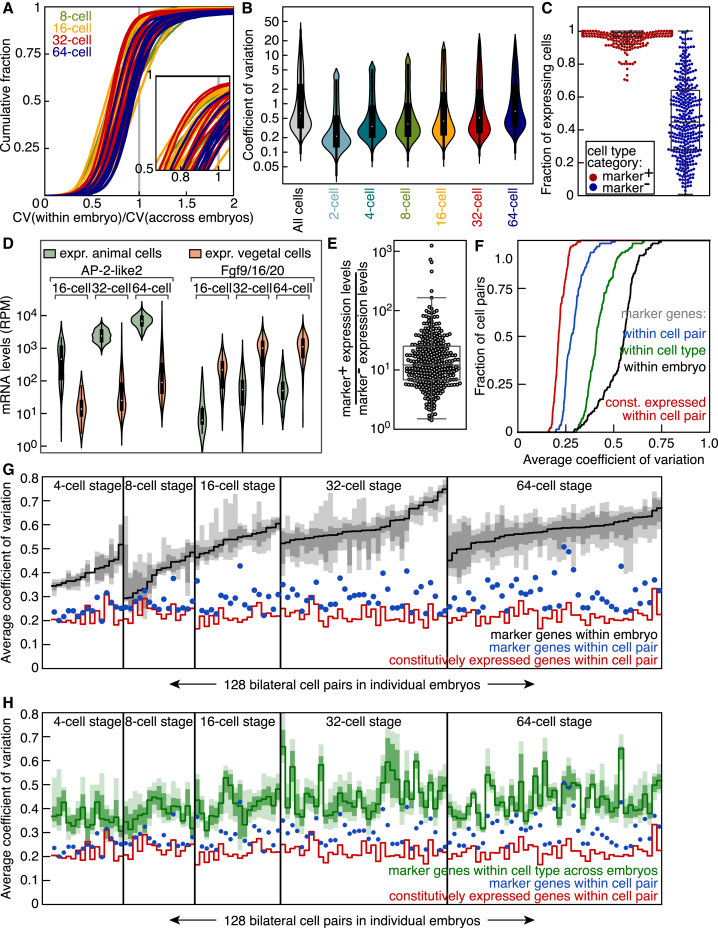
Precision of Gene Expression Regulation (A) Cumulative distribution of the coefficient of variation of expression levels within an embryo divided by the coefficient of variation of expression levels across embryos. Green, orange, red, blue lines are data from 8-, 16-, 32-, and 64-cell embryos, respectively. Inset, zoom of the plot. (B) Coefficient of variation of gene expression levels computed across single cells of embryos of the same stage. (C) Fraction of cells expressing marker genes that classify cell types (STAR Methods). (D) Violin plots of AP-2-like2 and Fgf9/16/20 expression levels in cells with detectable expression. (E) Comparison between marker gene expression levels in cells with detectable expression belonging to marker-positive or -negative cell types. (F) Cumulative distribution of the average coefficient of variation of expression levels of marker genes in bilaterally symmetric cell pairs (blue), in cells belonging to the same cell type (green), in cells belonging to the same embryo (black), and of the levels of genes with constitutive expression (red). (G and H) Average coefficient of variation of the expression levels of marker genes in two equivalent cells of the bilaterally symmetric embryo (blue dots) compared to cells randomly sampled in the same embryo ([G], black), compared to cells of the same cell type randomly sampled across embryos ([H], green), or compared to genes with constitutive expression in that same cell pair (red). Dark gray and green shade, interquartile range; light gray and green shade, 10%–90% range.

At the genome-wide level, we found a systematically lower degree of intraembryonic gene expression variability than cells randomly sampled from different embryos of the same stage (Figure 7A). Overall, this gene expression variability increased as development progressed (Figure 7B). We then explored the precision of gene expression regulation, taking advantage of both the high sequencing depth and our ability to unambiguously attribute single-cell expression profiles to a specific cell type within the embryo. We first identified 244 marker genes that were differentially expressed between cell types. We then queried the expression level of individual marker genes in cells identified as either marker-positive or -negative according to the average expression levels of the marker in the considered cell type. As expected, the vast majority of marker-positive cells expressed the respective marker genes; however, a small fraction of marker-negative cells also expressed individual marker genes (Figure 7C). For example, the animal pole marker AP-2-like2 was robustly expressed in animal cells but also found at 50-fold lower levels in a fraction of vegetal cells (Figure 7D). Conversely, the vegetal pole marker Fgf9/16/20 was expressed at 20-fold lower levels in a fraction of animal cells (Figure 7D). When looking at all the individual marker genes our unbiased approach had identified, we found a much larger magnitude of upregulation in marker-positive cell types than in marker-negative cell types (Figure 7E). These results suggest that although cells destined for a particular fate sporadically express marker genes of other lineages, this spurious transcription occurs only at 10-fold lower levels and from isolated loci. Thus, embryonic development can accommodate transcriptional noise at low levels.

Finally, the bilateral symmetry of *P. mammillata* embryos implies that similar cells are present on the left and right sides of individual embryos. A total of 23 cell types encompassed only 2 cells per embryo, and therefore, these cells could be unambiguously identified as bilaterally symmetric cell pairs. Our dataset had 128 such cell pairs (each pair originating from one embryo and belonging to the same cell type). We compared the levels of 305 marker genes with variable expression during embryogenesis with the levels of genes with constitutive expression in all cells. There was less variation in the expression of marker genes in bilaterally symmetric cell pairs from one embryo than in cells belonging to the same cell type but randomly sampled across embryos or cells randomly sampled within an embryo (Figure 7F). This variation was, in fact, comparable to the variation in the expression of genes with constitutive expression across all cells. Although bilaterally symmetric cell pairs were clear outliers compared to other cells of the same embryo (Figure 7G), the vast majority of cell pairs were more similar in their expression of marker genes compared to cells of the same cell type in other embryos (Figure 7H). In fact, ∼50% of cell pairs exhibited a precision in the expression of marker genes comparable to the one of housekeeping genes.

## Discussion

Interpreting single-cell transcriptome data is often hampered by the difficulty of matching individual gene expression profiles with the cell’s precise position, local environment, and function within the tissue architecture. Combining high depth scRNA-seq and high coverage of precisely staged individual embryos, we designed MorphoSeq, which consisted of computational frameworks that classify embryos into cell types without prior knowledge, reconstructed directly from its transcriptome the physical position and lineage history of each cell in an unbiased manner, and linked it to high-resolution 4D imaging data. We, thus, present the complete history of gene expression at the genome-wide level for every single cell and at every cell division in a developing embryo up to gastrulation.

The accurate reconstruction of lineage trees must account for the cell division history within particular cell types and the quantitative cell type composition of the embryo at different stages. Indeed, this consideration allowed us to map the sisters of the cells that belong to the germ cell lineage, whereas a gene-expression-based reconstruction ignoring the cell divisions occurring in the germ cell lineage would be erroneous. Precise mapping of cell division patterns can be obtained with light sheet microscopy that enables tracking cellular behaviors at the whole-embryo level (Keller et al., 2008, McDole et al., 2018). In addition, combining lineage tracing (McKenna et al., 2016) with sequencing (Raj et al., 2018, Wagner et al., 2018) will help address this aspect in vertebrate embryos.

During regulative developmental processes, such as vertebrate embryogenesis, cells will acquire their fate guided by environmental cues, and therefore, their gene expression will reflect their spatial position. Although recent advances in single-molecule fluorescent ISH techniques allow measurement of transcriptome-wide expression levels in tissues (Eng et al., 2019), a recent study demonstrated that it is possible to reconstruct *de novo* the spatial architecture of embryos or tissues directly from scRNA-seq data (Nitzan et al., 2019). ISH will, nevertheless, be required to position migrating cells within tissues.

In the case of a continuum of states during differentiation, the identification of individual cell types or differentiation intermediates would be problematic for any classification strategy. However, such cells usually possess a signature of the germ layer or tissue they belong to, whether in mouse (Peng et al., 2019, Sladitschek and Neveu, 2019), zebrafish (Wagner et al., 2018, Farrell et al., 2018), or *Xenopus* (Briggs et al., 2018). Examining genes with variable expression within that cell cluster would then reveal the existence of intermediates. Given our results on inter-embryo variability, such analyses would be greatly facilitated if performed on cells belonging to the same embryo.

The spatiotemporal atlas of single-cell gene expression linked to morphological features identified cell-adhesion molecules shaping the embryo. We found that cell-fate acquisition is driven by the rather simple mechanisms of cell-type-specific expression of transcription factors and the expression of signaling ligands in a spatially confined manner. Although the lineage reconstruction and the spatial mapping of cell types could largely be conducted using only three dimensions, classification of single-cell expression profiles was simplified using additional dimensions (fewer than 10 in total) that were specific to the subset of cells to classify. This indicated that ascidian development uses similar gene sets in a modular fashion to specify cell types along different lineages.

Previous studies have highlighted considerable noise in gene expression during development (Arias and Hayward, 2006), which might stem from (and readily be explained by) small differences in timing of embryogenesis between samples, as our analysis demonstrated. Conversely, pairs of bilateral symmetric cells that assume the same fate had remarkably similar gene expression profiles within a given embryo. This supports the notion that the coordinated gene regulatory networks underlying development yield a highly reproducible outcome (Levine and Davidson, 2005). Moreover, our high sequencing depth allowed us to capture the effect of transcriptional noise.

The canonical virtual embryo we report here (http://digitalembryo.org) is a valuable resource to mine the detailed molecular mechanisms that instruct the patterning of entire organisms or tissue architecture in a comprehensive manner. The ability to track systematically, quantitatively, and in a spatially resolved manner the genome-wide changes of gene expression of every cell at each cell division in embryos will usher in a new era in developmental biology. The MorphoSeq framework can be readily adapted to other stereotypically developing animals and might be applicable to more plastic embryonic development.

## STAR★Methods

### Key Resources Table

**Table:** 

REAGENT or RESOURCE	SOURCE	IDENTIFIER
**Chemicals, Peptides, and Recombinant Proteins**		

Proteinase K	Merck	1.24568.0100
FM4-64	ThermoFisher	T3166
Tn5 transposase	Protein Expression Core Facility, EMBL	N/A
Pronase	Merck	P5147
Sodium thioglycolate	Merck	T0632

**Critical Commercial Assays**		

NEBNext Ultra II DNA library preparation kit	NEB	E7645S
MirVana miRNA isolation kit	ThermoFisher	AM1560
TruSeq RNA Sample Preparation	Illumina	FC-122-1001
DIG RNA SP6/T7 labeling kit	Roche	11175025910
Rneasy mini kit	QIAGEN	74104
Superscript III First-Strand Synthesis SuperMix kit	ThermoFisher	18080-400

**Deposited Data**		

scRNA-Seq of *Phallusia mammillata* from 2-cell to 16-cell stage	This paper	ArrayExpress E-MTAB-6506
scRNA-Seq of *Phallusia mammillata* from 32-cell to 64-cell stage	This paper	ArrayExpress E-MTAB-6508
Gene expression profiles during *Phallusia mammillata* embryogenesis	This paper	ArrayExpress E-MTAB-6528
Genome sequencing of *Phallusia mammillata*	This paper	ArrayExpress E-MTAB-6530
single cell RNA-Seq data of *Ciona robusta* embryos	Treen et al., 2018	GSE110588

**Experimental Models: Organisms/Strains**		

Adult *Phallusia mammillata*	Roscoff Marine Station	N/A

**Oligonucleotides**		

Primer: PH domain of human PLCD1 Forward: AATACGCGTAACTCGAGATGGACTCGGGCC GGGACTTCC	This paper	N/A
Primer: PH domain of human PLCD1 Reverse: CTAGTCGACGATGTTGAGCTCCTTCAGGAA GTTCTGC	This paper	N/A
Primers for *in situ* probes, see Table S3	This paper	N/A

**Recombinant DNA**		

Genomic AHC0AAA267YK08 clone	Brozovic et al., 2018	N/A
Plasmid pRN3-MX	This paper	N/A
Plasmid pRN3-PH-Citrine	This paper	N/A
Plasmid pRN3-H2B-mCherry	This paper	N/A

**Software and Algorithms**		

Python 2.7.5	Python Software Foundation	https://www.python.org
Numpy 1.7.1	N/A	http://numpy.org
Scipy 0.12.0	N/A	http://scipy.org
Velvet	Zerbino and Birney, 2008	https://github.com/dzerbino/velvet
Oases	Schulz et al., 2012	https://github.com/dzerbino/oases
BLAST	Altschul et al., 1990	https://blast.ncbi.nlm.nih.gov
Bowtie 1.0	Langmead et al., 2009	https://bio.sourceforge.io/
ImageJ	Schneider et al., 2012	https://imagej.nih.gov/ij/
ImageMagick	ImageMagick Studio LLC	https://imagemagick.org/index.php
Chimera	Pettersen et al., 2004	http://www.cgl.ucsf.edu/chimera
ilastik	Sommer et al., 2011	https://www.ilastik.org
tulip	Auber et al., 2016	https://sourceforge.net/projects/auber/files/tulip/tulip-4.9.0/
IMARIS v. 8.4.1	Bitplane	https://imaris.oxinst.com
MARS	Fernandez et al., 2010	https://idp.nature.com/authorize?response_type=cookie&client_id=grover&redirect_uri=https%3A%2F%2Fwww.nature.com%2Farticles%2Fnmeth.1472
Amira v. 5.4.1 & v. 6.4.3	Thermo Scientific	https://thermofisher.com/amira-avizo
Blender	Blender Foundation	https://www.blender.org
MATLAB	Mathworks	https://www.mathworks.com/products/matlab
SCECTION	This paper	https://git.embl.de/neveu/morphoseq

**Other**		

Resource website for the scRNA-Seq of *P. mammillata* embryos	This paper	http://Digitalembryo.org

### Resource Availability

#### Lead Contact

Further information and requests for resources and reagents should be directed to and will be fulfilled by the Lead Contact, Pierre A. Neveu (neveu@embl.de).

#### Materials Availability

Plasmids generated in this study are available upon signature of an MTA.

#### Data and Code availability

The accession numbers for the sequencing results reported in this paper are ArrayExpress: E-MTAB-6506, E-MTAB-6508, E-MTAB-6528, E-MTAB-6530. They can be explored at http://digitalembryo.org. Code is available at https://git.embl.de/neveu/morphoseq.

### Experimental Model and Subject Details

Embryos were obtained from wild adult *Phallusia mammillata* animals that were procured from the Roscoff Marine Station (France). Embryos were allowed to develop in artificial sea water at 18^◦^C until collection for transcriptomics analysis. During imaging, embryos developed in artificial sea water as well. Embryo stages studied by scRNA-Seq were 2-cell, 4-cell, 8-cell, 16-cell, 32-cell and 64-cell stages. Imaging was performed up to 64-cell stage. *P. mammillata* individuals are hermaphodrites.

### Method Details

#### RNA-seq library construction

Adult *P. mammillata* animals were procured from the Roscoff Marine Station (France). RNA was extracted from *P. mammillata* staged embryos (staging was performed according to Hotta et al. 2007) using the MirVana kit (Ambion) following the manufacturer’s instructions. 30 barcoded stranded mRNA libraries representing 15 developmental stages were prepared using TruSeq RNA Sample Preparation (Illumina) following the manufacturer’s instructions. Libraries were sequenced in two runs on Illumina NextSeq 500 in the 75 bp single-end regime yielding 1.07 billion reads (161 Gbp total).

#### Genomic DNA extraction

Sperm from a single *P. mammillata* individual was lysed in 20 mL spooling buffer (75 mM NaCl, 25 mM EDTA pH 8.0, 1% SDS, 0.2 mg/ml Proteinase K) at 55^◦^C overnight with gentle shaking. 5 mL of saturated NaCl solution were added and the solution was mixed by gentle shaking till homogeneous. 25 mL of isopropanol were added and the solution was mixed by gentle shaking till homogeneous. The stringy DNA precipitate was fished out and washed in 70% ethanol. After removing the 70% ethanol, the DNA pellet was air-dried and resuspended in 10 mM Tris pH 8 at 55^◦^C with gentle shaking. A genomic DNA library was prepared using the NEBNext Ultra II DNA library preparation kit starting with 50 ng of sheared material following the manufacturer’s instructions. The library was run on Illumina MiSeq in the 250PE regime yielding 18.4 million reads (9.2 Gbp).

#### Transcriptome assembly

Our *P. mammillata* transcriptome assembly involves a strategy relying on consensus building in peptide space from a large number of assemblies followed by several refining iterations. For each mRNA library, transcripts were assembled using Velvet (Zerbino and Birney, 2008) and Oases (Schulz et al., 2012) using 31-mers. Further processing of the Oases output was carried out using custom Python scripts. Transcript models were translated in the three forward reading frames, keeping the longest peptide for each transcript. Peptides were sorted according to their C-termini, enabling us to discard partially assembled proteins. This yielded a collection of 350,000 different C-termini. Practically, peptides of different sizes for each C terminus were found in many assemblies. For each C terminus, we retained the longest > 99% identical peptides found in more than one assembly, reasoning that they most likely corresponded to the full-length protein. This set of consensus peptides was aligned to *Ciona robusta* (formerly known as *Ciona intestinalis* type A but mostly referred to as *Ciona intestinalis*) protein sequences using BLAST (Altschul et al., 1990). We retained peptide models with a *C. robusta* hit at most 25% longer than the query and an alignment score greater than 1.5 times the target length. A Bowtie index was built for the transcripts corresponding to these “high confidence” peptides. mRNA reads were aligned to this index using Bowtie (Langmead et al., 2009) with default parameters. For each sample, reads with a match were discarded and the remaining reads were used for a new round of assembly and peptide consensus building. The entire procedure was iterated a third time. This aforementioned strategy efficiently assembled transcripts up to 20 kb. In order to identify potentially larger genes, peptide models with matching overlap were further assembled. This led to the full unsupervised assembly of the largest gene in vertebrate genomes Titin with a transcript 77.5 kb long encoding for 25,560 amino acids, representing 189 exons spanning a genomic locus of 140 kb. Peptide models were annotated using the best *C. robusta* BLAST hit. Altogether, our *P. mammillata* transcriptome assembly comprised 14,203 transcript models representing 12,945 gene models. We validated a set of transcripts by amplifying them from cDNA, cloning in a custom plasmid (Sladitschek and Neveu, 2015a) and Sanger sequencing of the insert. Assembled transcripts are in the process of being deposited at the European Nucleotide Archive.

#### Genome assembly

As a quality control for our transcriptome assembly and to get the reconstruction of transcripts with introns, we independently assembled *P. mammillata* genome using custom Python scripts. We matched both ends from paired-end reads of short inserts in order to generate longer sequences that will be used for further assembly. This generated ∼18 million 250-450 bp long sequences. Sequences corresponding to ribosomal RNAs and genomic repeats were identified from their high coverage and assembled. Reads mapping to these regions were discarded leaving us with ∼16 million reads for assembly. To minimize assembly errors, we again adopted a strategy that relied on consensus building from independent assemblies. We started from batches of 3 million reads representing an average of 10-fold coverage. Reads were chosen randomly and extended by overlap matching. Contigs from the different assemblies were then matched to each other and validated by mapping transcripts to them. Transcripts that were not fully mapped without gaps were used as scaffolds. Seeds in exons were extended by overlap matching for the assembly of the introns.

#### Single-cell collection

*P. mammillata* embryos were dissociated in 0.2% pronase (Sigma), 1% sodium thioglycolate (Sigma), 50 mM NaOH in calcium-free artificial sea water for 20 minutes and individual dissociated embryos were transferred to calcium-free artificial sea water in imaging plates (Mat-tek) coated with a solution of 0.1% PFA and 0.1% gelatin. Dissociated embryos were kept on ice before single-cell collection and during collection. Individual cells were manually collected in 0.3 μL with pipet tips coated with a solution of 0.1% PFA and 0.1% gelatin and cells were directly lysed in 4 μL of lysis+dNTP+oligodT mix (2 μL of lysis buffer – 0.2% Triton X-100+RNase inhibitor–, 1 μL of 10 μM oligo-dT primer – sequence 5′AAGCAGTGGTATCAACGCAGAGTACT30VN –, 1 μL of 10 mM dNTP mix) according to the Smart-Seq2 protocol (Picelli et al., 2014). 1084 cells were collected as follows: eight 2-cell embryos, eight 4-cell embryos, eight 8-cell embryos, eleven 16-cell embryos, 380 cells from fourteen 32-cell embryos, 31 cells from one 44-cell embryo and 385 cells from eight 64-cell embryos. All embryos up to the 16-cell stage were complete, 32-cell embryos were 72% to 97% complete and 64-cell embryos were 48% to 94% complete.

#### Single-cell RNA-Sequencing

cDNA from single cells was prepared following the Smart-Seq2 protocol (Picelli et al., 2014) using 18 pre-amplification PCR cycles. Smart-Seq2 was found to be the most sensitive and accurate method with the smallest dropout rate in a recent survey of six scRNA-Seq methods (Ziegenhain et al., 2017). 500 pg of cDNA was then used for the tagmentation reaction using a home-made Tn5 transposase (Hennig et al., 2018). All samples were processed with the same batch of reagents. Barcoded libraries were pooled in 12 batches of 84 to 96 samples. Libraries were sequenced in twelve runs on Illumina NextSeq 500 in the high density 75 bp single-end regime yielding 6.65 billion reads total (498.9 Gbp total).

#### scRNA-seq analysis and quality control

We built a Bowtie index for the assembled transcripts. mRNA reads were aligned to this index using Bowtie (Langmead et al., 2009) allowing up to 3 mismatches. The Bowtie output was parsed to count the number of reads aligning to each transcript model. 3.35 billion reads mapped to our transcriptome assembly, with an average of 3.09 million mapped reads per cell. We removed 42 cells with < 100,000 mapped reads, leaving us with 1042 cells out of 1084. On average, there were 10,173 ± 655 detected transcripts per cells with at least 5,500 detected transcripts in the cell with the smallest sequencing depth that was retained for further analysis. In comparison, we detected for 11,179 ± 290 transcripts in bulk mRNA-Seq at the similar embryonic stages. Notably, 8,542 ± 272 genes had expression levels > 4 RPM. In comparison, we detected for 8,392 ± 131 transcripts with expression levels > 4 RPM in bulk mRNA-Seq at 32- and 64-cell stages. Thus, our scRNA-Seq depth was comparable to bulk mRNA-Seq depth.

#### Normalization

The very high scRNA-Seq depth circumvented the problem of many zero counts commonly encountered in scRNA-Seq. Read counts were normalized in two steps to correct for different sequencing depth. In a first step, samples were coarsely normalized by multiplying read counts by a factor to have the same total number of reads across all samples. We identified 654 genes with a minimum expression > 64 RPM across all samples (including the ones with low sequencing depth). A fine normalization factor was determined by matching the log-transformed read counts of these 654 genes to the identity line for samples pairwise. For each sample, read counts were then multiplied by this fine normalization factor. It should be noted that each sample was normalized by multiplying read counts by a single factor that was independent of the read count. Read counts were not normalized by the transcript length for individual genes as we were solely interested in relative expression changes across samples. Technical batch effects were not detectable. However, we could detect batch effects due to biological variability. Some genes had several highly polymorphic alleles that were only found in the embryos of some individuals. The sum of the read counts mapping to the different alleles was used for downstream analysis. In addition, the coefficient of variation of gene expression increased as development progressed. This was probably due to the apportioning of the mRNA pool deposited in the oocyte between sister cells upon cell division. Moreover, the coefficient of variation of gene expression in individual embryos was smaller compared to the coefficient of variation across embryos of the same stage (see Noise analysis paragraph below). This was probably due to slight differences in the quantity of mRNAs deposited in individual oocytes.

#### Identification of maternal factors

Before the onset of zygotic genome activation, different expression profiles can only be generated by selective RNA inheritance or degradation. Such a process should occur in an even number of cells (due to the bilateral symmetry of ascidians) and reproducibly in individual embryos. We therefore sought to identify genes that would single out a similar fraction of cells in the different embryos that were profiled at the same stage. Expression levels were clipped to a minimum of 1 RPM. We retained genes with maximal expression > 16 RPM (reads per million mapped reads) in at least one cell and at least a 4-fold difference in expression between cells. Expression levels were log transformed. For each stage and each gene, cells were rank ordered according to expression levels. Difference in expression between subsequent ranks was computed. The index of the largest difference was used to classify the cells in two categories: the cells rank ordered below that index belonged to the first category and the cells rank ordered above the index belonged to the second category. At the 4-cell stage, only 10 genes (out of 3354 candidates) split the cells in two categories, sampling individual embryos evenly. Remarkably, the categories defined independently by these 10 genes were identical (picking the same 15 or 16 cells out of 31 randomly has a probability of 6.65 10^−9^). At the 8-cell stage, 10 (out of 3354 candidates) genes classified cells into two categories: one category with lower expression comprising three quarter of the cells and one with higher expression comprising one quarter of the cells. Like for the 4-cell stage embryos, the same 15 cells were singled out by all these 10 genes (there are 1.22 10^14^ 15-combinations of cells from 63 cells), picking exactly two cells in each 8-cell embryo (and one cell in an 8-cell embryo for which only seven cells passed quality control). Furthermore, these genes were identical to the ones identified at the 4-cell stage. We then determined if these genes would stratify cells in 16- and 32-cell stage embryos. Indeed, they singled out at most two cells per embryo for an average of two cells per embryo (when taking into account sampling error) for both stages. To further identify genes which mRNAs would be co-segregated in the same cells, we looked for genes with a differential enrichment. For each gene and each individual embryo, we computed its expression enrichment in the cells singled out by the 10 genes compared to all the other cells of the embryo by making the ratio of its mean expression levels in each category. We defined as maternal factors genes that had an increased expression in the cells singled out by the 10 genes for all the embryos profiled from 4- to 16-cell stage (27 embryos total). 27 genes and some mitochondrial transcripts fulfilled that criterion.

#### Cell type classification

We reasoned that genes that classify cell types should have markedly different expression levels in single cells of a given embryo. In addition, cell types should have an even number of cells in a complete embryo due to the bilateral symmetry of *P. mammillata*. We thought to identify cell types for individual embryonic stages.

##### 8-cell stage

8-cell stage embryos have in theory at most 4 cell types. The expression of maternal factors that are asymmetrically inherited singled out 2 cells in each 8-cell stage embryo. Using our bulk mRNA-Seq data, we retained genes with maximal expression > 1 RPM in 4- and 8-cell embryos and a 2-fold difference in expression between the two stages. This list of 24 genes was narrowed down by keeping the genes with maximal expression > 1 RPM and at least a 4-fold difference in expression between cells of each embryo for all the eight 8-cell embryos profiled. This left us with 4 potential classifiers. As there are only 2 cells with high maternal factor expression per embryo, these cells cannot be further subclassified. Thus, classification among cells with high maternal factor expression had to be significantly different than the classification of all the cells of 8-cell stage embryos (χ^2^-test with Benjamini-Hochberg correction for multiple hypothesis testing, p < 0.05). 2 genes out of 4 passed p < 0.05, the transcription factor Fkh and an ankyrin repeat and SAM domain-containing protein. Fkh was expressed at higher levels in 4 cells per 8-cell embryo and not detected in the remaining 4 cells (including the cells with high maternal factor expression). The other gene was not expressed in the cells with high maternal factor expression.

##### 16-cell stage

16-cell stage embryos have in theory at most 8 cell types. The expression of maternal factors that are asymmetrically inherited singled out 2 cells in each 16-cell stage embryo. Using our bulk mRNA-Seq data, we retained genes with maximal expression > 1 RPM in 4- and 16-cell embryos and a 2-fold difference in expression between the two stages. We clipped the expression data at a minimum of 1 RPM and we used the same strategy and criteria to find potential classifier genes as for 8-cell embryos. After the χ^2^-test, we had 48 candidate classifiers. Using these candidate classifiers and the 27 maternal factors, we performed non-negative matrix factorization (NMF) with a varying number of metaprofiles (Neveu et al., 2010) on individual embryos. Gene expression levels were log2-transformed. For each gene, the mean expression level across the cells of a given embryo was subtracted and the variance was normalized to one. We found that whereas two metaprofiles split the embryos in two clusters of 8 cells each, three metaprofiles split the embryos in 5 clusters, 3 comprising 4 cells and 2 comprising 2 cells. 4 metaprofiles did not increase the number of clusters but instead lead to a degraded consensus clustering compared to 3 metaprofiles. Clustering was determined according to Brunet et al. (2004): for each number of metaprofiles, we determined the consensus matrix as the average connectivity matrix over 100 clustering runs.

#### Cell type classification by SCECTION

To implement Single-Cell Expression Classification Through Iterations Of NMF (SCECTION), we used Non-negative Matrix Factorization (NMF) as an unsupervised way to cluster the single-cell expression profiles.

##### SCECTION framework

We clipped the expression data at a minimum of 1 RPM. We retained genes with maximal expression > 4 RPM in at least one cell and a 16-fold difference in expression in the set of cells to be classified. Gene expression levels were log2-transformed. For each gene, the mean expression level across the set of cells to be classified was subtracted and the variance was normalized to one. NMF with two metaprofiles was computed as described (Lin, 2007). Clustering was determined according to Brunet et al. (2004): we determined the consensus matrix as the average connectivity matrix over 50 clustering runs. Subsets of cells belonging to a cluster with more than 75% clustering consensus were used for a subsequent SCECTION iteration. The iterative process stops when no gene satisfies the differential expression condition within the set of cells to be classified or when SCECTION outputs no cluster with more than 75% consensus clustering.

##### Cell type matching across embryos

Within embryo, average expression profiles were computed for each cell type using log-transformed expression values. For each embryo, the levels of each gene were rescaled by subtracting by their mean expression level and rescaled by their standard deviation. This removed embryo-specific expression trends. The expression profile for cell type *j* in embryo *k* consisted of log-transformed expression values $\left\{x_{j,i}^{k}\right\}$ for the associated genes $\left\{g_{i}\right\}$. Relatedness between cell types in different embryos *k* was determined using average linkage clustering using the Euclidean distance between the average expression profiles. $\left\{x_{j}^{k}\right\}$ were matched with the closest $\left\{x_{j'}^{k'}\right\}$ profiles. Cell types were expected to be found across several embryos according to sampling rules, that is cell types found in a single embryo were merged to their closest matching type (corresponding to the subset of cells that generated that specific cell type in the last SCECTION round) within that embryo.

##### Application of SCECTION

We applied SCECTION to the eleven 16-cell stage embryos. SCECTION split the 16-cell stage embryo into 5 cell types, 3 comprising 4 cells and 2 comprising 2 cells that were similar to the cell types recovered by running NMF with 3 metaprofiles on individual embryos. The first SCECTION round separated animal from vegetal cells, the second round split each pole into the A/a and B/b lineages while the third round of SCECTION distinguished B5.1 and B5.2 in the B lineage. SCECTION applied to 32-cell stage embryos identified 8 cell types: 6 cell types in the vegetal pole (2 cell types in the A lineage, 4 cell types in the B lineage) and 2 cell types in the animal pole. SCECTION applied to 64-cell stage embryos identified 18 cell types: 13 cell types in the vegetal pole (6 cell types in the A lineage, 7 cell types in the B lineage) and 5 cell types in the animal pole (2 cell types in the a lineage, 3 cell types in the b lineage). Final attribution of single cells to individual cell types was manually validated. *In situ* hybridization data in *Ciona* (Imai et al., 2004) supported both our classification and the constellation of genes expressed in a given cell type.

#### Lineage tree reconstruction

Mother-daughter relationships were established by assessing the expression of the genes that classified the mother cells after one cell division. Using our bulk mRNA-Seq data, we retained genes with maximal expression > 1 RPM in 4-cell embryos and the embryonic stage of the mother cells and a 2-fold difference in expression between the two stages. Average expression profiles were computed for each cell type using log-transformed expression values. For each stage, the levels of each gene were rescaled by subtracting by their mean expression level and rescaled by their standard deviation. This removed stage-specific expression trends. The expression profile for cell type *j* at stage *k* consisted of log-transformed expression values $\left\{x_{j,i}^{k}\right\}$ for the associated genes $\left\{g_{i}\right\}$. Relatedness between cell types at stage *k* and the subsequent embryonic stage *k* + 1 (representing one cell division) was determined using average linkage clustering using the Euclidean distance between the expression profiles. $\left\{x_{j}^{k}\right\}$ were matched with the closest $\left\{x_{j}^{k+1}\right\}$ profiles until the sum of the cells contained in the matching profiles was equal to twice the number of cells present in cell type $\left\{x_{j}^{k}\right\}$ (to account for the doubling of cells through cell division). This is in effect a parsimony argument minimizing the number of cell type changes between mother and daughter cells. The lineage tree reconstructed from our scRNA-Seq data was fully compatible with the actual cell lineage tree of *P. mammillata*.

#### Dimensionality reduction

The lineage tree of different cell types provides a natural way to embed the data. One local axis was the developmental progression of individual cells from mother to daughter cell types. Position along this local axis was computed by projecting the gene expression profile of an individual cell on the direction defined by the average expression profiles of the mother cell type and the daughter cell type the cell belongs to. Arrangement on a second local dimension was done by determining nearest neighbors as measured by the Euclidean distance in the space of genes with differential expression between mother and daughter cell types.

#### RNA velocity analysis

RNA velocity was performed following La Manno et al. (2018) under the constant velocity assumption. Reads aligning to introns or intron-exon junctions were considered to originate from unspliced RNA molecules (reads mapping to individual exons were not counted in that category as they cannot be unambiguously attributed to an unspliced molecule). For individual genes, the normalized degradation rate γ was computed using the set of cells with the top decile of expression. The velocity *v* was then computed as follows *v* = *u* − γ *s* where *u* is the unspliced read counts and *s* the spliced read counts (La Manno et al., 2018). Values for *v* are smaller than the unspliced read counts (which can be 1000-fold smaller than the spliced read counts due to the compactness of ascidian genomes), we therefore scaled *v* by 1/γ to extrapolate the spliced read counts (indeed at steady state *u* ∼γ*s*; La Manno et al., 2018). Neither genes nor cells were pooled to estimate the velocity of individual cells. To visualize RNA velocities, the extrapolated spliced read counts were projected on the relevant space (Axis16_1_ and Axis16_2_ or the two-dimensional space of the lineage tree) and were the tips of the velocity arrows. In the case of projection on Axis16_1_ and Axis16_2_, we used all genes that were used for the principal component analysis to compute these two axes. In the case of the two-dimensional space of the lineage tree, we used genes with a difference of expression greater than 4-fold between mother and daughter cell types.

#### Spatial mapping of single-cell transcriptomes

##### Embryonic axis determination at 16-cell stage

We relied on principal component analysis (PCA) to find the directions which explained most of the variance in gene expression between single cells of individual 16-cell embryos. PCA was carried out as described (Sladitschek and Neveu, 2015b). Genes with 4-fold expression changes within an embryo were kept for subsequent analysis. Expression profiles were log-transformed and the principal components were computed using gene expression profiles of cells of a single embryo. The expression profile for cell *j* consisted of log-transformed expression values $\left\{x_{i}^{j}\right\}$ for the associated genes $\left\{g_{i}\right\}$. PCA determined principal components (PCs) $\left\{X_{\alpha }\right\}$ corresponding to the set of eigenvalues $\left\{\lambda_{\alpha }\right\}$ of the covariance matrix. PCs $\left\{X_{\alpha }\right\}$ were rescaled by the square root of the associated eigenvalue $\lambda_{\alpha }$. The first PCs were rotated to remove the trends across PCs between cells within the embryonic poles defined by the first round of SCECTION. This determined a new coordinate system with axes Axis16_β_ orthogonal to each other. For cell *j*, we plotted the projection on the α^th^ Axis16: $\left(x^{j}-\bar{x}\right)\cdot Axis16_{\alpha }$ where $\bar{x}$ was the mean expression profile averaged on all cells from the embryo. The eleven 16-cell stage embryos retrieved exactly the same directions (with occasional swaps of direction along one axis).

##### Mapping of single cells at 32-cell stage

Log-transformed transcriptomes of single cells of individual 32-cell stage embryos were projected on the Axis16 coordinate system.

##### Mapping of cell types

Log-transformed expression levels were averaged among all cells of a given cell type and were projected on the Axis16 coordinate system.

##### Finer mapping at 64-cell stage

The vegetal pole of 64-cell embryos consists of 13 cell types and the Axis16 coordinate system cannot resolve the spatial relationship of some of the sister cell types. We reasoned that the axes that map the vegetal pole of 32-cell embryos might discriminate cells at the 64-cell stage. We defined two axes which shared the same global direction: the one that classifies the daughters of B6.1 compared to the ones of B6.2 and the one that classifies the daughters of A6.1&3 compared to the ones of A6.2&4. The B lineage could be perfectly mapped on Axis16_1_ and this first axis with the exception of the germ cell B7.6. The A lineage could be mapped using the second axis and the direction of the most remaining variance.

#### Asymmetric cell divisions

Before the onset of zygotic transcription, a partition bias of one RNA will lead to a relative increase and decrease in the levels of this particular RNA in the two sister cells. In the absence of zygotic transcription: *v*_1_/*v*_2_ = (*x*_0_ − *x*_2_)/(*x*_1_ − *x*_0_) where *v*_1_ and *v*_2_ are the volumes of the sister cells, *x*_0_, *x*_1_ and *x*_2_ are the concentrations of the given RNA in the mother cell and sister cells respectively. Using the levels of the maternal factors PEM-1, Vasa and Distal-less Dll-B, we found that B5.2 was 3 times smaller than its sister B5.1.

#### Comparison with Ciona expression profiles

We used the analyzed *Ciona robusta* single-cell RNA-Seq data from Treen et al. (2018) as provided by the authors on GEO with accession number GSE110588.

#### Noise analysis

In order to assess the variability of single-cell gene expression, we analyzed independently each embryo we collected. We considered only genes with a maximal expression greater than 16 RPM in the embryo and computed the coefficient of variation of their expression across the cells from the embryo. For the same gene set, a mean coefficient of variation was estimated from hundred random samplings of a similar number of cells from the embryos of the same stage. To find candidate marker genes, we retained genes with maximal expression > 4 RPM in at least one cell type and at least a 4-fold difference in mean expression between cell types with high and low expression. High and low expressing cell types were then classified in either marker-positive or marker-negative categories. The fraction of cells expressing the marker gene was then determined for each of the two categories. We then assessed the levels of the marker genes in cells with detectable expression for each of the two categories. This allows to investigate two different scenarios of gene expression regulation: (i) a Boolean case where there is a single expression ON state for marker genes and the control point would be the fraction of cells expressing the gene, (ii) cells can control the expression levels if the gene is not in an OFF state.

#### Analysis of gene upregulation coordination

We retained for analysis genes with maximal expression > 4 RPM and that were upregulated more than 4-fold between the mother and daughter cell types of the specific cell type considered. Log-transformed expression levels were rescaled for each gene to have a mean expression equal to zero in the mother cells and a mean expression equal to one in the daughter cells. Individual cells were then sorted according to the sum of these rescaled expression levels. To compare the coordination of gene expression between embryos, the average expression per cell type was computed within each individual embryo for each gene. Log-transformed average expression levels were rescaled for each gene to have a mean expression equal to zero in the mother cell type and a mean expression equal to one in the daughter cell types. Individual embryos were sorted according to the sum of these rescaled expression levels for each individual cell type and the consensus order was retained. Embryos were randomized to test for the significance of the correlation of expression levels between cell types (p < 10^−52^ for 16-cell stage cell types, p < 10^−20^ for 32-cell stage cell types, and p < 10^−5^ for 64-cell stage cell types, Kolmogorov-Smirnov test).

#### Temporal model of gene expression variation

Log-transformed expression levels in individual cells were normalized to have a mean expression of 0 in the mother cell type and 1 in the grand-daughter cell type. The expression of an individual gene *i* was considered to vary linearly in time *t* with rate $\alpha_{i}$ in the form of $\left\{0\;if\;t<t_{0}^{i};\alpha_{i}\left(t-t_{0}^{i}\right)\;if\;t\geq t_{0}^{i}\right\}$, where $t_{0}^{i}\;$is the time of the expression ramp-up. According to this model, the expression in cell *j* captured at time $t_{j}$ will be $\left\{0\;if\;\;t_{j}<t_{0}^{i};\alpha_{i}\left(t_{j}-t_{0}^{i}\right)\;\;if\;t_{j}\geq t_{0}^{i}\right\}$. Measured expression levels in individual cells were then adjusted using two parameters per gene $\left\{\alpha_{i},t_{0}^{i}\right\}$ and one per cell $\left\{\;t_{j}\right\}$. To assess the quality of the fit, we used the residuals between the predicted values (computed using the parameter sets $\left\{\alpha_{i},t_{0}^{i}\right\}$ and $\left\{\;t_{j}\right\}$) and the measured values for individual genes.

#### Constructs for live imaging

MluI and XhoI restriction sites were inserted between the 5′ and 3′ β-globin UTRs in a pRN3 expression vector (Lemaire et al., 1995) containing PH-GFP (a gift of Alex McDougall) to yield pRN3-MX and make it compatible with the MXS-chaining technique (Sladitschek and Neveu, 2015a). Citrine and the PH domain of human PLCD1 (subcloned using primers Forward: 5′-AATACGCGTAACTCGAGATGGACTCGGGCCGGGACTTCC and Reverse: 5′-CTAGTCGACGATGTTGAGCTCCTTCAGGAAGTTCTGC) were successively chained in pRN3-MX yielding pRN3-PH-Citrine. H2B-Cherry was chained in pRN3-MX to give pRN3-H2B-Cherry.

#### Embryo imaging

The embryos were microinjected prior to fertilization with mRNA encoding for PH-Citrine and H2B-mCherry using phenol red as microinjection dye. Microinjected embryos were incubated for ∼3 hours at 18^◦^C prior to fertilization. The embryos were imaged from the 2-cell stage up to the gastrulation period using multi-view light-sheet microscopy (MuVi-SPIM) (Krzic et al., 2012). We used a MuVi-SPIM configuration with two Nikon 10X, 0.3 NA water-dipping objectives for the illumination and two Olympus 20X, 1.0 NA water-dipping objectives to detect emitted photons and a 300 mm tube lens to provide a 33.3X magnification of the sample. The embryos were imaged across four orthogonal angles –for each time point the sample was rotated 0^◦^ and 90^◦^– with simultaneous dual illumination and dual acquisition, using electronic confocal slit detection (de Medeiros et al., 2015) adjusted to the illumination beam size. Stacks of images every 1 μm were acquired every 2 minutes with Hamamatsu ORCA-Flash4.0 V2 sCMOS cameras.

#### Image processing

Multi-angle data was fused to produce isotropic resolution images using bead-based affine registration to yield a single high-quality 3D dataset for each time point. Fluorescence images were rendered using Chimera (Pettersen et al., 2004). Cell lineage extraction was based on nuclei tracking. Nuclei probability maps were created using ilastik pixel classification (Sommer et al., 2011). Tracking was done using the ilastik semi-automated method “Manual Tracking Workflow.” Lineage representation was created using the data visualization software tulip (Auber et al., 2016). Nuclei cell identification was done by 3D visualization of the segmented and ilastik tracked labeled nuclei in IMARIS (version 8.4.1). Digital 3D representations of the cellular morphology of each cell in the embryo were created using machine learning (pixel classification) to extract membrane probability maps with ilastik (Sommer et al., 2011). Single time point segmentations were done through membrane-based 3D watershed segmentation with the algorithm MARS (Fernandez et al., 2010) on the membrane probability maps and creating segmented embryo surface representations in AMIRA (versions 5.4.1 and 6.4.3) or the open source software Blender (https://www.blender.org). Time-course membrane-based segmentations were done using 3D-seed watershed segmentation with MATLAB image analysis libraries using the segmented and tracked nuclei as seeds. Tesselation of the segmented embryos was performed using the marching cubes algorithm (Lorensen and Cline, 1987). The marching cube algorithm was kindly provided by the Kreshuk laboratory (EMBL, Heidelberg). The tessellated surfaces generated for each individual cell were smoothed for visualization purposes using the open source software Blender. The 3D virtual *in situs* in http://digitalembryo.org result from a color-coded expression level projection on the surface of the tessellated embryo representations.

#### Creation of animated 4D renderings

The membrane signal was segmented in individual frames as described (Sladitschek and Neveu, 2015b). Cells were matched between frames using the Hungarian algorithm in order to obtain a 3D reconstruction. Individual time points were registered by translation to keep the center of mass of the entire embryo invariant. Tracking cells from one time point to the next was done by matching the position of a cell’s center of mass at time *i* with the corresponding cell at time *i* − 1. Grayscale 3D renderings of the segmented membrane signal were created using the VolumeViewer plugin of ImageJ (Schneider et al., 2012). 4D renderings were created by coloring the grayscale 3D renderings according to cell lineage, cell types or gene expression levels using a custom python script. Gene expression levels were interpolated between stages using a logistic function. Annotation of python-generated colored time frames and conversion to gif format was performed using ImageMagick for the 12,945 genes.

#### Apical surface and protocadherin expression

SPIM images were used to compute for each cell its volume, the area of its surface and its apical surface (i.e., the surface in contact with the environment), the length of the apical boundary between individual cells. The computed cadherin contact strength for cell *i* is defined as $\sum_{j}\min\left(C_{i},C_{j}\right)\times l_{ij}$, where $C_{i}$ and $C_{j}$ are the cadherin expression levels in cells *i* and *j*, $l_{ij}$ the length of the apical boundary between cells *i* and *j*.

#### Perturbation of cell-cell adhesion

*Phallusia* embryos were treated with calcium free artificial sea water for ≈12 min during late 32-cells stage prior to imaging with light sheet microscopy in calcium-free artificial sea water containing the lipophilic dye FM464 (6 μM) for membrane labeling.

##### Whole mount *in situ* hybridization

Total mRNA was extracted from 50 μL of Phallusia embryos at 112-cells stage using the QIAGEN Rneasy mini kit. A cDNA library was produced using oligodT and random hexamers and the Superscript III First-Strand Synthesis SuperMix kit (Invitrogen). The AHC0AAA267YK08 clone from the publicly available *Phallusia mammillata* EST clone collection (Brozovic et al., 2018) was used as a template for generating the TTF1 *in situ* probe. Templates for probe synthesis were generated by PCR amplification from the cDNA library introducing a T7 promoter in the reverse primers either for the primary reaction from cDNA (for MyoD and Nodal) or in a subsequent amplification (for the other genes). Primers used for AP-2-like2, Fkh, FoxD-a/b, Macho1, MyoD, Nodal, Tbx6b, Wnt3 and ZicL can be found in Table S3. The TTF1 EST clone was linearized by EcoRI digestion. All *in situ* probes were made using the DIG RNA SP6/T7 labeling kit (Roche) and purified with the QIAGEN RNeasy RNA purification kit. Whole mount *in situ* hybridizations were performed as described (Christiaen et al., 2009).

### Quantification and Statistical Analysis

Statistical tests were computed using the Python SciPy module. The statistical tests used were two-sided and are indicated in the text or in the figure legends. The number of samples is indicated in the figure legends. Significance was set at p < 0.05. Normality was assessed by the Shapiro-Wilk test.

### Additional Resources

Sequencing results can be explored at http://digitalembryo.org.
