# Supplementary material for: MorphoSeq: Full Single-Cell Transcriptome Dynamics Up to Gastrulation in a Chordate
Source: Cell. 2020 May 14;181(4):922–935.e21. doi: 10.1016/j.cell.2020.03.055 (PMC7237864; doi:10.1016/j.cell.2020.03.055)
Supplement: Document S1. Tables S1–S3 [file mmc1.pdf]

Cell, Volume *181*

Supplemental Information

MorphoSeq: Full Single-Cell Transcriptome Dynamics Up to Gastrulation in a Chordate

Hanna L. Sladitschek, Ulla-Maj Fiuza, Dinko Pavlinic, Vladimir Benes, Lars Hufnagel, and Pierre A. Neveu

Embryo label	Embryo stage	Collected cell #
02cell08111	2-cell	2/2
02cell08112	2-cell	2/2
02cell08113	2-cell	2/2
02cell08114	2-cell	2/2
02cell08115	2-cell	2/2
02cell08116	2-cell	2/2
02cell08117	2-cell	2/2
02cell08118	2-cell	2/2
04cell08111	4-cell	4/4
04cell08112	4-cell	4/4
04cell08113	4-cell	4/4
04cell08114	4-cell	4/4
04cell08115	4-cell	4/4
04cell08116	4-cell	4/4
04cell08117	4-cell	4/4
04cell08118	4-cell	4/4
08cell08111	8-cell	8/8
08cell08112	8-cell	8/8
08cell08113	8-cell	8/8
08cell08114	8-cell	8/8
08cell08115	8-cell	8/8
08cell08116	8-cell	8/8
08cell08117	8-cell	8/8
08cell08118	8-cell	8/8
16cell06081	16-cell	16/16
16cell06082	16-cell	16/16
16cell06201	16-cell	16/16
16cell09091	16-cell	16/16
16cell09092	16-cell	16/16
16cell09093	16-cell	16/16
16cell09094	16-cell	16/16
16cell09095	16-cell	16/16
16cell09096	16-cell	16/16
16cell09097	16-cell	16/16
16cell09098	16-cell	16/16
32cell06091	32-cell	28/32
32cell06092	32-cell	23/32
32cell06093	32-cell	30/32
32cell06191	32-cell	28/32
32cell06193	32-cell	26/32
32cell06211	32-cell	23/32
32cell06212	32-cell	26/32
32cell06213	32-cell	31/32
32cell09040	32-cell	28/32
32cell09041	32-cell	25/32
32cell09043	32-cell	26/32
32cell09044	32-cell	31/32
32cell09045	32-cell	27/32
32cell09046	32-cell	28/32
64cell06081	64-cell	32/64
64cell06201	64-cell	47/64
64cell06202	64-cell	31/64
64cell08071	64-cell	46/64
64cell08072	64-cell	53/64
64cell09081	64-cell	59/64
64cell09082	64-cell	57/64
64cell09083	64-cell	60/64

Supplementary Table S1. Summary of single-cell collection for scRNA-Seq. Related to Figure 1.

Cell type	Total cell #	Corresponding cells
AB2	16	AB2
A3	16	A3
B3	15	B3
A4.1&a4.2	32	A4.1, a4.2
B4.1	15	B4.1
b4.2	16	b4.2
A5.1-2	43	A5.1, A5.2
B5.1	20	B5.1
B5.2	20	B5.2
a5.3-4	43	a5.3, a5.4
b5.3-4	42	b5.3, b5.4
A6.1&3	47	A6.1, A6.3
A6.2&4	48	A6.2, A6.4
B6.1	24	B6.1
B6.2	18	B6.2
B6.3	21	B6.3
B6.4	21	B6.4
a6.5-8	95	a6.5, a6.6, a6.7, a6.8
b6.5-8	95	b6.5, b6.6, b6.7, b6.8
A7.1-2	27	A7.1, A7.2
A7.3&7	25	A7.3, A7.7
A7.4	12	A7.4
A7.5	10	A7.5
A7.6	13	A7.6
A7.8	11	A7.8
B7.1-2	26	B7.1, B7.2
B7.3	12	B7.3
B7.4	13	B7.4
B7.5	10	B7.5
B7.6	7	B7.6
B7.7	8	B7.7
B7.8	10	B7.8
a7.9-10	16	a7.9, a7.10
a7.11-16	79	a7.11, a7.12, a7.13, a7.14, a7.15, a7.16
b7.9	10	b7.9
b7.10	11	b7.10
b7.11-16	66	b7.11, b7.12, b7.13, b7.14, b7.15, b7.16

Supplementary Table S2. Cell types identified by scRNA-Seq and corresponding *P. mammillata* cells. Related to Figure 4.

Primer name	Sequence
AP-2-like2 Forward	AATACGCGTTTCCCAGTTTTGCGCTGCTTCATCA
AP-2-like2 Reverse	CTAGTCGACTGACAGAACTCCAACTCCCCGCTAC
Fkh Forward	AATGCTAGCATGATGTTGTCGTCTCCCCCATCAAAG
Fkh Reverse	CTAGGTACCTTAGTTGGCCGGTACGCACCCTTG
FoxD-a/b Forward	AATACGCGTGAATGACAGTTCAGACCTACGGACAC
FoxD-a/b Reverse	CTAGTCGACTTACGTTGTGCGTCCAAAACAAGGCC
Macho1 Forward	AATACGCGTCCTACCCCGGCTGTGGAAAGGTATT
Macho1 Reverse	CTAGTCGACAGGGGTGTGGGTGATTGAAGTTGGT
MyoD Forward	TCATGACCGTCGCCGCGCGGCCA
MyoD Reverse	CTAATACGACTCACTATAGGGCATAGTTCGTCGGTGATGC
Nodal Forward	CTATGGATATGACACAAGTATCGTTCTGC
Nodal Reverse	GATCCTAATACGACTCACTATAGGGTTATCGACATCCACATTCT
Tbx6b Forward	AATACGCGTTCGCAGTTTGAACCATCTTCTGCCG
Tbx6b Reverse	CTAGTCGACTGTATCGCTTTGTGAAGGCTGGTGC
Wnt3 Forward	AATGCTAGCATGAATTTAGTTGGTTCTGTGGCGGTCTG
Wnt3 Reverse	CTAGGTACCCGTCACTTGCACGTGTGTTCCTCG
ZicL Forward	CTAGTCGACCGAGTTTCTTTTCCGGCAGCTCACA
ZicL Reverse	AATACGCGTACGTGACCACCGTTTTGGTTAGGTG

Supplementary Table S3. Primer sequences. Related to STAR Methods.
